# How Dendrites Affect Online Recognition Memory

**DOI:** 10.1371/journal.pcbi.1006892

**Published:** 2019-05-03

**Authors:** Xundong Wu, Gabriel C. Mel, D. J. Strouse, Bartlett W. Mel

**Affiliations:** 1 School of Computer Science and Technology, Hangzhou Dianzi University, Hangzhou, China; 2 Computer Science Department, University of Southern California, Los Angeles, CA, United States; 3 Physics Department, Princeton University, Princeton, NJ, United States; 4 Biomedical Engineering Department and Neuroscience Graduate Program, University of Southern California, Los Angeles, CA, United States; Cold Spring Harbor Laboratory, UNITED STATES

## Abstract

In order to record the stream of autobiographical information that defines our unique personal history, our brains must form durable memories from single brief exposures to the patterned stimuli that impinge on them continuously throughout life. However, little is known about the computational strategies or neural mechanisms that underlie the brain's ability to perform this type of "online" learning. Based on increasing evidence that dendrites act as both signaling and learning units in the brain, we developed an analytical model that relates online recognition memory capacity to roughly a dozen dendritic, network, pattern, and task-related parameters. We used the model to determine what dendrite size maximizes storage capacity under varying assumptions about pattern density and noise level. We show that over a several-fold range of both of these parameters, and over multiple orders-of-magnitude of memory size, capacity is maximized when dendrites contain a few hundred synapses—roughly the natural number found in memory-related areas of the brain. Thus, in comparison to entire neurons, dendrites increase storage capacity by providing a larger number of better-sized learning units. Our model provides the first normative theory that explains how dendrites increase the brain’s capacity for online learning; predicts which combinations of parameter settings we should expect to find in the brain under normal operating conditions; leads to novel interpretations of an array of existing experimental results; and provides a tool for understanding which changes associated with neurological disorders, aging, or stress are most likely to produce memory deficits—knowledge that could eventually help in the design of improved clinical treatments for memory loss.

## Author summary

Humans can effortlessly recognize a pattern as familiar even after a single presentation and a long delay, and our capacity to do so even with complex stimuli such as images has been called "almost limitless". How is the information needed to support familiarity judgements stored so rapidly and held so reliably for such a long time? Most theoretical work aimed at understanding the brain's one-shot learning mechanisms has been based on drastically simplified neuron models which omit any representation of the most visually prominent features of neurons—their extensive dendritic arbors. Given recent evidence that individual dendritic branches generate local spikes, and function as separately thresholded learning/responding units inside neurons, we set out to capture mathematically how the numerous parameters needed to describe a dendrite-based neural learning system interact to determine the memory's storage capacity. Using the model, we show that having dendrite-sized learning units provides a large capacity boost compared to a memory based on simplified (dendriteless) neurons, attesting to the importance of dendrites for optimal memory function. Our mathematical model may also prove useful in future efforts to understand how disruptions to dendritic structure and function lead to reduced memory capacity in aging and disease.

## Introduction

**Fig 1:**
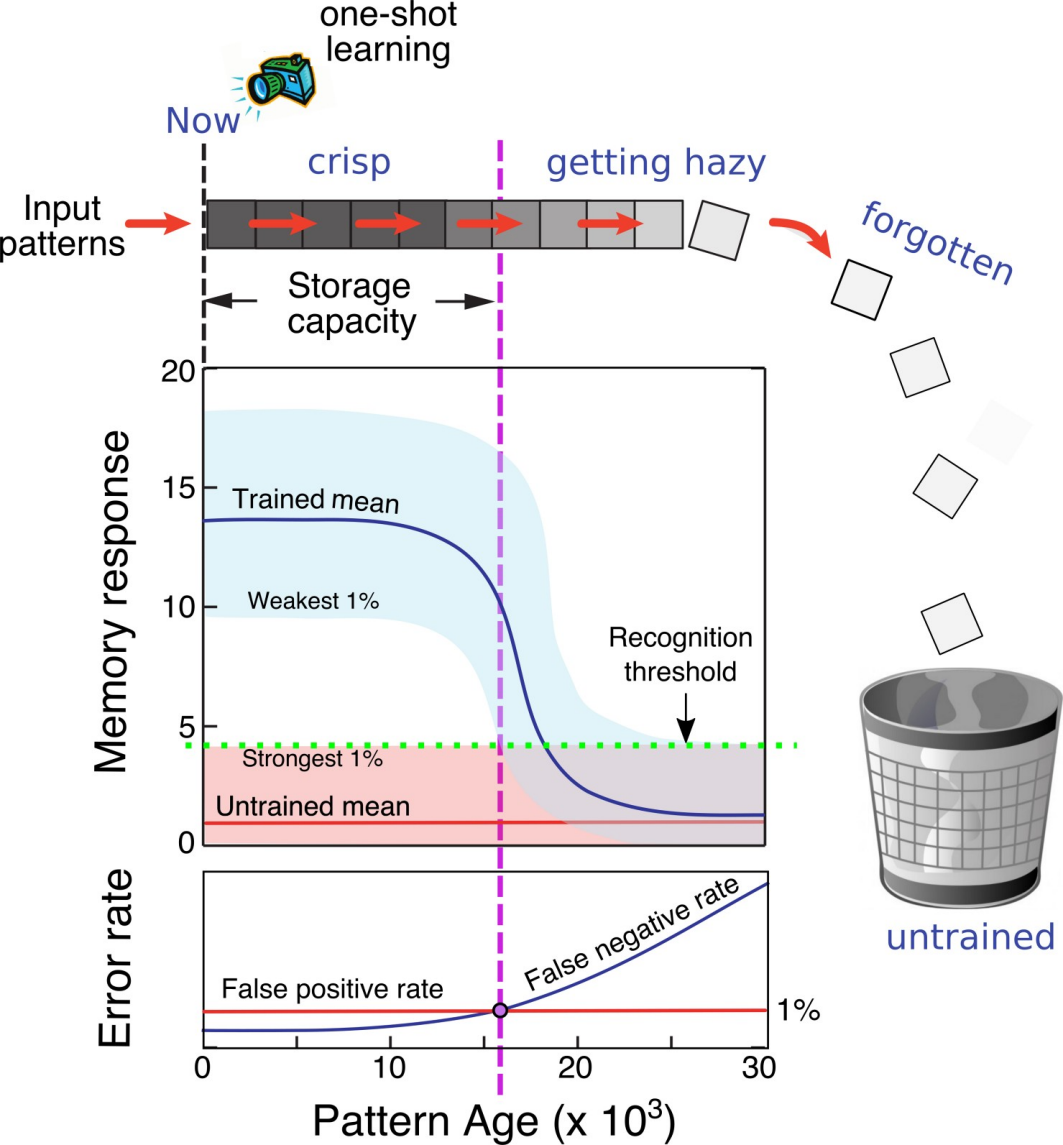
Online learning in a familiarity-based recognition memory. Novel patterns are streamed continuously into the memory and "one-shot" learned. Memory responses to trained patterns are shown as a distribution in light blue; distribution of responses to untrained (random) patterns is shown in light red. Recognition threshold separating the two distributions is shown as a green dashed line, set to produce a 1% false positive error rate. As stored patterns approach the end of their lifetimes, their traces decay and begin to merge with the untrained background distribution, leading to an increase in the false negative error rate (i.e. "misses"). Capacity is operationally defined as the pattern age at which the miss rate (averaged over all patterns up to that age) becomes unacceptably high (chosen to be 1% here).

To function well in a complex world, our brains must somehow stream our everyday experiences into memory as they occur in real time. An “online” memory of this kind, once termed a “Palimpsest” [1], must be capable of forming durable memory traces from a single brief exposure to each incoming pattern, while preserving previously stored memories as long and faithfully as possible (Fig 1). This combined need for rapid imprinting and large capacity requires that the memory system carefully manage both its learning and forgetting processes, but we currently know little about how these processes are implemented and coordinated in the brain.

A number of quantitative models have been proposed for palimpsest-style online memories, and have addressed a variety of different issues, including: how memory capacity scales with network size, how metaplastic learning rules can increase memory capacity, and the tradeoff between initial trace strength and memory lifetimes [1–8]. A few studies with a more empirical focus have addressed the biological mechanisms underlying recency vs. familiarity memory [9]; the coordination of online learning with long-term memory processes; and the details of memory-related neuronal response properties during online learning tasks [10–12].

Nearly all previous models of online learning have assumed that the neurons involved in memory storage are classical "point neurons”, that is, simple integrative units lacking any representation of a cell’s dendritic tree. This simplification is notable, given the now substantial evidence from both modeling and experimental studies that dendritic trees are powerful, functionally compartmentalized information processors that can augment the computing capabilities of individual neurons in numerous ways [7,13–59].

Beyond their contributions to the computing functions of neurons, it is also increasingly apparent that dendrites help to organize and spatially compartmentalize synaptic plasticity processes [7,40,60–86].

Thus, given that dendrites can act as both signaling *and* learning units within a neuron, it is important to understand how having dendrites could affect the brain’s online learning and memory processes. In this paper, we focus on the role that dendrites may play in familiarity-based recognition, a function most closely associated with the perirhinal cortex [87,88].

Here, we introduce a mathematical model that allows us to calculate online storage capacity from the underlying parameter values of a previously proposed dendrite-based memory circuit [7]. The model includes biophysical parameters (dendritic learning and firing thresholds, network recognition threshold), wiring-related parameters (number of axons, number of dendrites, number of synapses per dendrite), and input pattern statistics (pattern density, noise level) (see Table 1). As an example of the model’s use, we study the interactions between memory capacity, dendrite size, and pattern statistics, and cross-check the results using full network simulations. We found that dendrites containing a few hundred synapses (as opposed to a few tens or a few thousand) maximize storage capacity, providing the first normative theory that accounts for the actual sizes of dendrites found in online memory areas of the brain.

**Table 1:** List of parameter categories, and specific parameters, used in the analysis and simulations.

Parameter Categories (in order of increasing flexibility)	Parameters
Task parameters (fixed across simulations)	*θ*_*±*_
Network parameters (mostly fixed across simulations)	*N*_*A*_, *N*_*S*_, *f*_*s*_, *D*_*j*_
Signal parameters (explored across simulations)	*f*_*A*_, *N*_*burst*_, *P*_*burst*_
Threshold parameters (optimized per simulation)	*θ*_*F*_, *θ*_*Lpost*_, *θ*_*Lpre*_, *θ*_*R*_
Main parameters of interest (optimized per simulation)	*K*, *M*
Learned parameters (altered during learning events)	*w*_*ij*_, *α*_*ij*_

## Results

**Fig 2:**
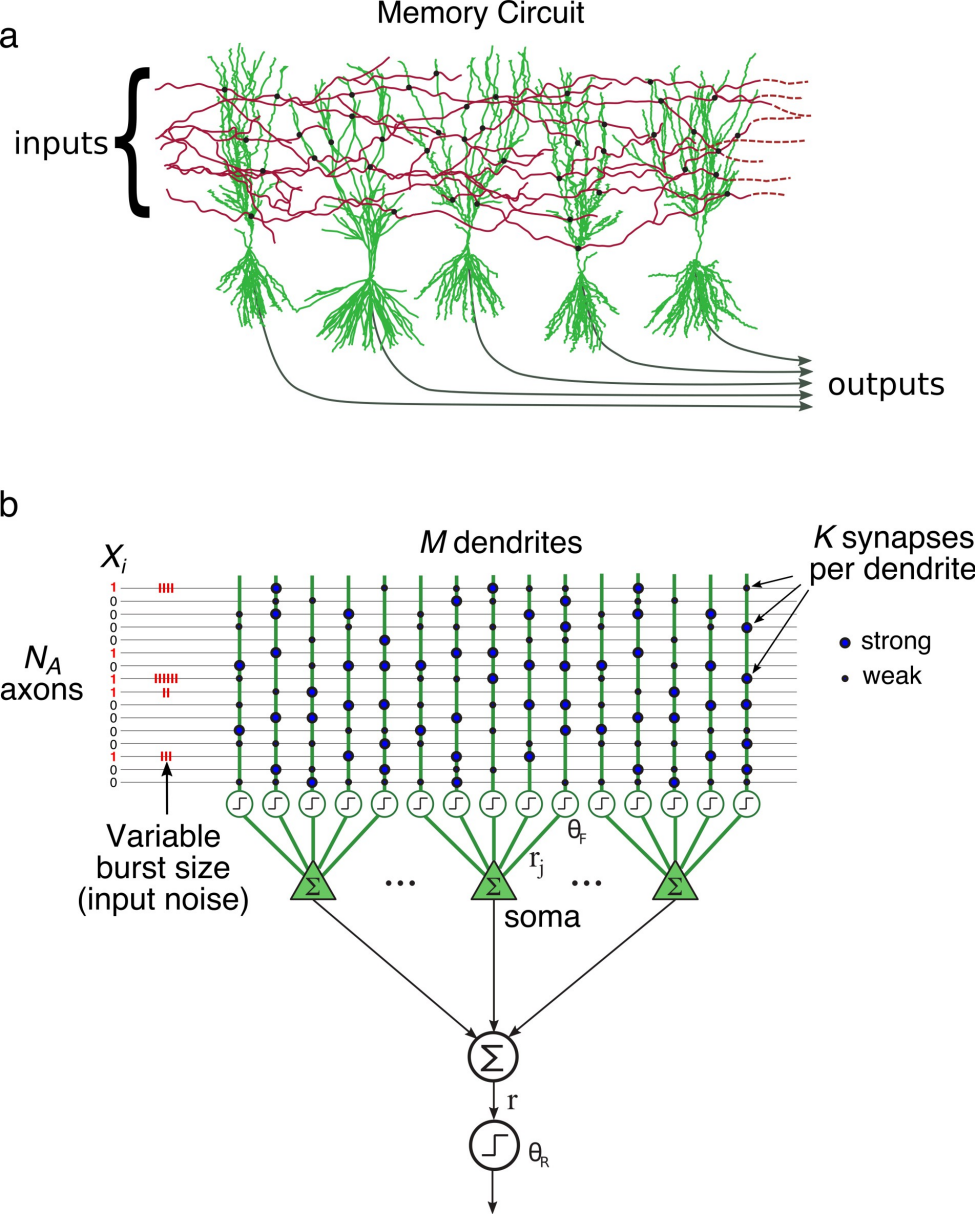
Architecture of the memory circuit. (**a**) A set of input axons makes sparse random contacts with the dendrites of a set of post-synaptic neurons. Only a subset of axons and neurons are shown. Patterns are stored by modifying synaptic weights, indicated by black circles. (**b**) Abstraction of the memory network shown in (a). Neurons are assumed to linearly combine dendritic outputs, so that the overall network response *r* is effectively a sum over all dendritic responses. The assumption of linear summation at the soma is included for simplicity, but is of little practical importance: the probability that any given dendrite fires in response to a particular pattern is very low, so that a neuron almost never contains more than a single firing dendrite (making the summation rule moot).

We modeled the memory network depicted in Fig 2a, consisting of a set of axons that form sparse random connections with the dendrites of a population of target neurons. An “infinite” sequence of random binary patterns is presented by the axons to the dendrites, each one causing one-shot changes to certain synapses within the network, where the goal of the network is to respond weakly to any pattern on its first presentation, and strongly for as long as possible to patterns that have been previously experienced. We define capacity as the number of consecutive training patterns stretching from “now” back into the past that can be classified as familiar with a low false negative (i.e. “miss”) rate, while maintaining a low false-positive (i.e. “false alarm”) rate to randomly drawn distractors (Fig 1).

### The network

The network structure and plasticity rules have been previously described in [7], but are repeated here for clarity. A population of neurons with a total of M separately thresholded dendrites receives inputs from *N*_*A*_ input axons (Fig 2b). Each dendrite receives K synaptic contacts randomly sampled from the *N*_*A*_ axons, for a total number of synapses $N_{S}\;=\;M∙K$. The connectivity matrix is assumed to be fixed.

Input patterns are binary-valued vectors *x* = {*x*_*1*_,…,*x*_*NA*_} for which component $x_{i}$ is 1 if the $i^{th}$ axon is “firing” and 0 otherwise. We quantify density/sparsity of the patterns by the fraction of axons $f_{A}$ firing in each pattern; the value of $f_{A}$ ranged from 0.008 to 0.18 in this study, as we found empirically in previous work that sparse patterns maximize capacity in this type of memory [7]. To model a biologically realistic form of input variability, we assumed that each active axon ($x_{i}\;=\;1$) produces a burst of spikes, where the number of spikes in the burst is drawn from a binomial distribution with mean *$\mu_{burst}\;=\;N_{burst}\cdot P_{burst}\;=\;4$* spikes/burst. $P_{burst}$ ranged from 1 (no noise) to 0.4 (high noise), with $N_{burst}$ varying inversely. Inactive axons ($x_{i}\;=\;0$) were assumed to produce no spikes. We denote the noisy spike count version of an input component ${\tilde{x}}_{i}\;\sim \;x_{i}∙Binom(N_{burst},P_{burst})$.

Synapses are characterized by both a weight $w_{ij}$, where the subscript indicates a connection between axon i and dendrite j, and an additional scalar parameter $\alpha_{ij}$, representing the synapse’s “age”. The weight of each synapse is binary-valued, and can change between weak (*w* = 0) and strong (*w* = 1) states when the dendrite containing the synapse undergoes a learning event; the conditions that trigger a learning event are discussed below. The age variable at each synapse tracks the number of learning events that have occurred in the parent dendrite since the synapse last participated in learning.

Two different measures of a dendrite’s activation level determine how the dendrite responds to an input, and whether it undergoes a learning event. The “presynaptic” activation measure is based on the activity levels of the set of axons *D*_*j*_ that make contact with the *j*th dendrite

$$a_{pre}^{\left(j\right)}=\sum_{i\epsilon D_{j}}{\tilde{x}}_{i}.$$

In words, $a_{pre}^{\left(j\right)}$ is the total number of presynaptic spikes arriving at all the synapses impinging on the $j^{th}$ dendrite, regardless of their postsynaptic weights, and is thus a measure of the maximum response the dendrite *could* muster to that input pattern assuming all of the activated synapses were strong (w=1).

The more conventional “postsynaptic” activation level takes account of the synaptic weights in the usual way:

$$a_{post}^{\left(j\right)}=\underset{i\epsilon D_{j}}{\sum }w_{ij}\cdot {\tilde{x}}_{i}.$$

When the postsynaptic activation level exceeds the “firing” threshold $\theta_{F}$, the dendrite is said to fire, that is, generates a response *r*_*j*_ = 1. The responses of all dendrites within a neuron sum linearly to produce the neuron’s response (Fig 2b), and the responses of all neurons in the network sum linearly to produce the overall network response r. The overall response of the network can therefore be written directly as a sum over all the M dendritic responses:

$$r=\underset{j\epsilon [1,M]}{\sum }r_{j}$$

so that the network can be viewed as a single “super neuron” with M dendrites.

Finally, an input pattern is classified as “familiar” if $r\geq \theta_{R}$, and “novel” if $r<\theta_{R}$, where *θ*_*R*_ is the recognition threshold (Fig 2b).

### The synaptic learning rule

The goal of learning is to ensure that learned patterns going back as far as possible in time produce suprathreshold network responses $\left(r\geq \theta_{R}\right)$, while randomly drawn patterns do not. Learning of any given pattern occurs in only the small fraction of dendrites that cross both the presynaptic and postsynaptic learning thresholds ($a_{pre}^{\left(j\right)}>\theta_{Lpre}$ and $a_{post}^{\left(j\right)}>\theta_{Lpost}$). When this occurs, a “learning event” is triggered in the dendrite, and all active synapses belonging to that dendrite “learn”, as follows. If an active synapse is currently in the weak state, it is “potentiated” (i.e. both strengthened and “juvenated”: $w_{ij}\to 1,\;\alpha_{ij}\to 0$), or if it is already in the strong state, then it remains strong but is juvenated ($w_{ij}\;=\;1,$ $\alpha_{ij}\to 0$). All strong synapses in the dendrite that are *not* active during the learning event remain strong but grow older ($w_{ij}\;=\;1,\alpha_{ij}\to \alpha_{ij}+1)$. Thus $\alpha_{ij}\;$counts the number of learning events that have occurred in the dendrite since the synapse last learned, and thus represents the age of the most recent information that that synapse is involved in storing. Note that a synapse’s age variable counts learning events within its parent dendrite only, and any given dendrite learns only rarely, so the counter need have only a small number of distinct values, on the order of ~12 under the simulation conditions explored in this paper. To maintain a constant fraction of strong synapses (we used $f_{s}\;=\;0.5$), and thereby to prevent saturation of the memory, in each dendrite undergoing learning, a number of strong synapses are depressed ($w_{ij}\to 0$) equal to the number of weak synapses potentiated during that learning event. A key feature of the learning rule is that the synapses targeted for depression are those that learned least recently (i.e. having the largest values of $\alpha_{ij}$), so that the information erased during depression is the “oldest” stored information. This “age-ordered depression” strategy substantially increases online storage capacity [5], especially in a 2-layer dendrite-based memory where the very sparse use of synapses during pattern storage gives each strong synapse, and the information it represents, the opportunity to grow old [7].

### Calculating memory capacity

**Fig 3:**
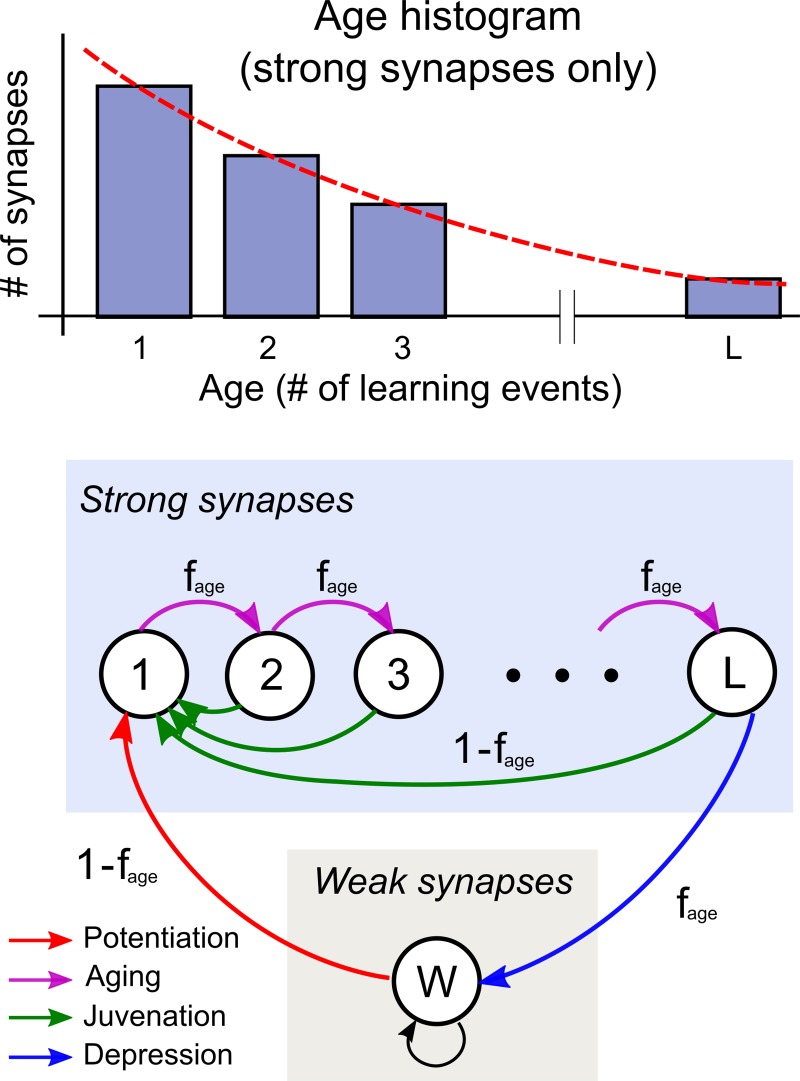
Synapse ages and the associated markov model. Conceptual bar graph at top shows steady state probabilities of synapse ages within a typical dendrite; age is counted in learning events. Markov model shows the L age states of a strong synapse and the one weak state, with transitions of four types as indicated in the legend. Transition probabilities are shown on the arrows.

One of the key quantities involved in calculating storage capacity is L, the length of the age queue within a dendrite (see Fig 3). An approximate expression for L is given here; the derivation can be found in the Methods.

$$L=\left[\frac{\log(1-f_{S})}{\log\left(1-\frac{\theta_{Lpre}}{K\cdot \mu_{burst}}\right)}-1\right]$$ (1)

L is a measure of the time a pattern feature persists in a dendrite, and given that age queues progress at roughly equal rates in all the dendrites involved in storing a pattern, it also effectively measures a pattern’s lifetime in memory–counted in units of dendritic learning events. L can be understood intuitively through an oversimplified example: If 10 synapses are strengthened on a dendrite during a learning event, and there are 120 strong synapses on the dendrite, then L would be ~12. That is, after ~12 learning events have elapsed since a pattern was first stored, the 10 synapses involved in storing the pattern are now the oldest on the dendrite and must be depressed, and the memory is lost. The actual expression for L is more complex as it takes into account the fact that strong synapses do not inexorably progress to the ends of their age queues–they can be rejuvenated one or more times during the course of their lifetimes, in which case the same strong synapse participates in the representation of more than one pattern.

To convert from L to a number of training patterns, we must multiply L by the approximate number of patterns per dendritic learning event, or “learning interval” $\frac{1}{P_{L}}$, where $P_{L}$ is the probability that an arbitrary dendrite learns a particular pattern. This gives an expression for capacity:

$$C\approx \frac{L}{P_{L}}=\;\frac{1}{P_{L}}\;\left[\frac{\log(1-f_{S})}{\log\left(1-\frac{\theta_{Lpre}}{K\cdot \mu_{burst}}\right)}-1\right]$$ (2)

Although $P_{L}$ is conceptually simple, its expression is complicated since it depends on pattern density, noise level, two learning thresholds, dendrite size, and $f_{S}$, and so it is omitted here for clarity (see in the Methods section for the full expression and some discussion).

### Calculating memory capacity

The expression for C measures how long patterns persist in memory, but a different calculation is needed in order to predict the memory’s recognition performance, that is, the false positive and false negative error rates $\epsilon_{+}$ and $\epsilon_{-}$ that we can expect to obtain during a pattern’s lifetime. These error rates depend on the separation of the distributions of responses to trained vs. untrained patterns (Fig 1). These two distributions can be computed from the network parameters to determine whether the allowable error rate tolerances $\theta_{+}$ and $\theta_{-}$ will be met during the lifetime calculated in Eq 2 (see Methods).

### Determining optimal dendrite size

How can the expression for online storage capacity (Eq 2) be exploited? Given that one of the unique features of our model is that dendrites are the learning units, we used the model to determine how capacity varies with dendrite size, which in turn allows us to determine the optimal dendrite size. In particular, we asked: for a fixed total number of synapses in the memory network ($N_{S}\;=\;M∙K$), if the goal is to maximize online storage capacity, is it better to have many short dendrites (i.e. large M, small K), a few long dendrites (small M, large K), or something in between? Furthermore, how does the optimal dendrite size vary with properties of the input patterns, such as pattern density and input noise level? To address these questions, we fixed network parameters $N_{s}\;$and $f_{s}$ and then for varying combinations of the pattern-related parameters ($f_{A},\;N_{burst},\;P_{burst})$, we computed C as a function of dendrite size K, using values of the learning, firing, and recognition thresholds ($\theta_{Lpost},\;\theta_{Lpre},\;\theta_{F},\;\theta_{R}$) optimized for each value of K through a semi-automated grid search. The “optimal” dendrite size under a particular set of input conditions was the value of K that maximized capacity, subject to the constraint that immediately after training, responses to trained patterns were strong enough, and responses to random patterns were weak enough, that both the false positive ($\epsilon_{+}$) and false negative ($\epsilon_{-}$) error rates fell below specified tolerances (we used 1% for both). Note that though K appears explicitly only once in Eq 2, as a result of the capacity optimization process, all of the thresholds, and consequently $\theta_{Lpre}$ and $P_{L}$ in Eq 2 depend implicitly on K. The net effect of these dependencies is analyzed in detail in the sections below on penalties for long and short dendrites.

**Fig 4:**
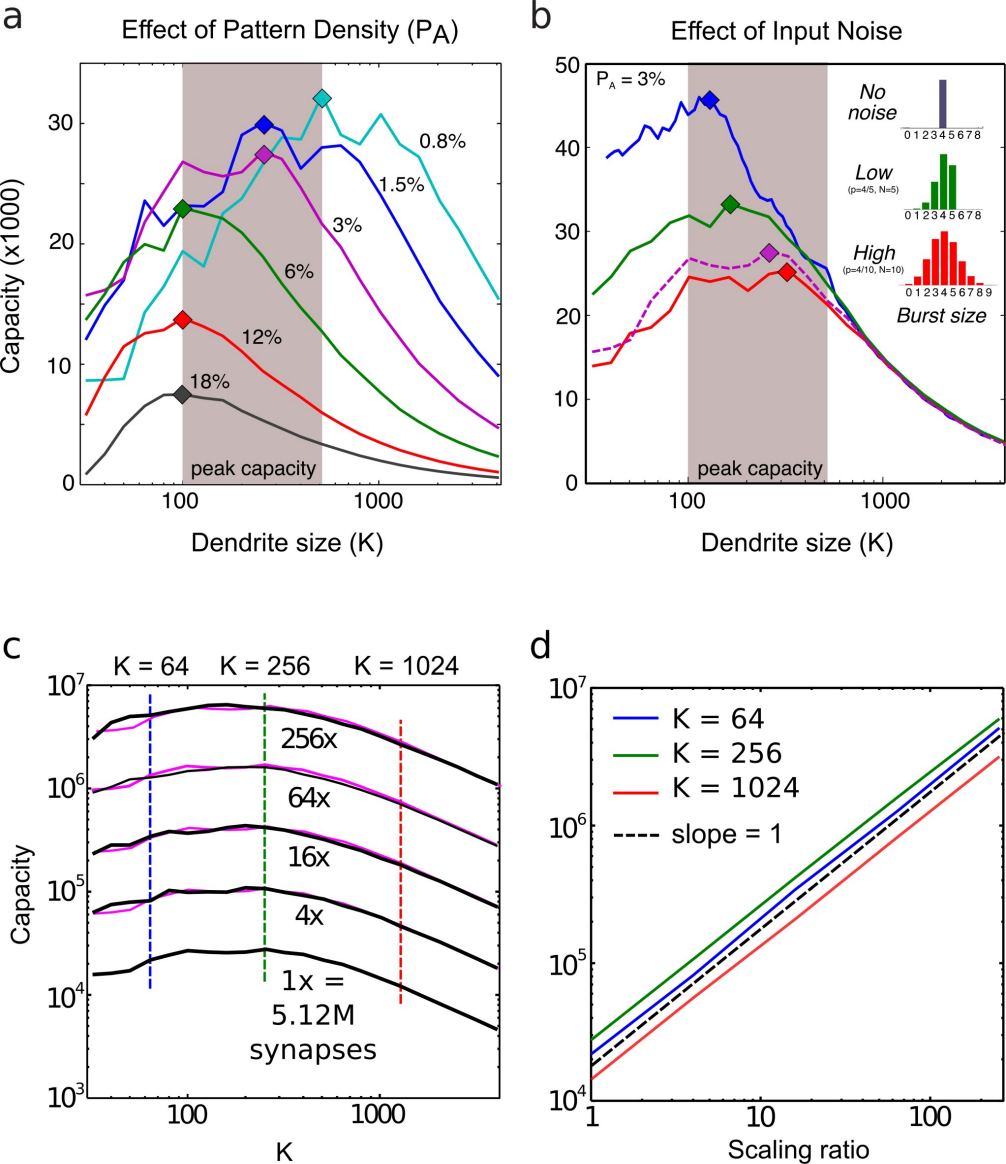
Capacity as a function of dendrite size. (**a**) Capacity curves are plotted for pattern densities ranging from 0.8% to 18%. Dendrite size is plotted on a log scale. Peak capacities lie in the range of 100–500 synapses per dendrite. Sparser patterns lead to a preference for longer dendrites and produce higher storage capacities (but not because sparse patterns contain less information–see main text and S1 Text). “Jagged” capacity curves for short dendrites and/or low pattern densities are due to a combination of (1) small numbers of synapses active per dendrite, and (2) quantization of dendritic learning and firing threshold to integer values, which may be optimal for some dendrite sizes but suboptimal for others. (**b**) Capacity curves for increasing values of input burst noise. Distributions of spike counts per burst are shown as bar plots. Dashed magenta curve corresponds to the solid magenta curve in (a); this curve represented a medium noise condition with $P_{burst}\;=\;4/7,\;N\;=\;7$. Noisier inputs reduce capacity, and lead to a preference for longer dendrites. (**c**) Capacity curves for increasing number of synapses. Capacity is plotted on a log scale. Magenta curves are vertically shifted (therefore scaled) versions of the 1x curve, to show that the dependence of storage capacity on dendrite size remains stable over a wide range of network scales. (**d**) Capacity scales nearly linearly for increasing network sizes, shown for three dendrite sizes (corresponding to vertical dashed lines in c). Dashed diagonal shows slope of 1 (representing perfect linear scaling) for comparison.

**Fig 5:**
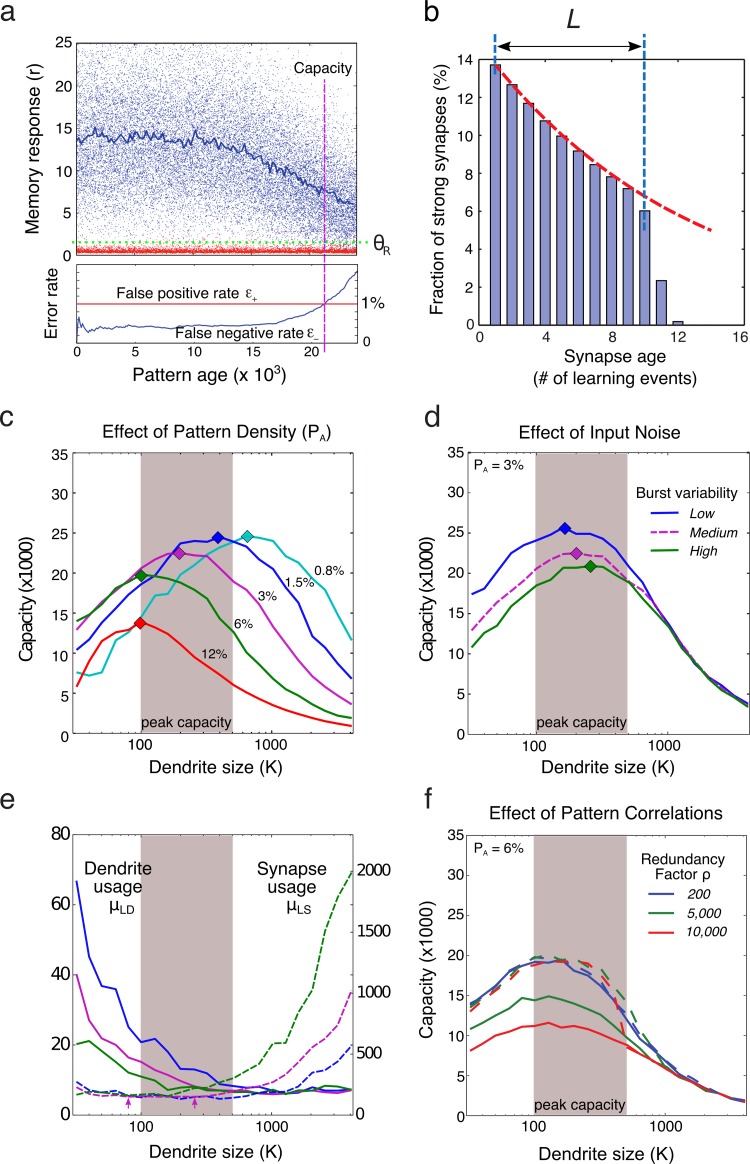
Validating the analytical model with full network simulations. (**a**) Dots show trace strengths of individual trained (blue) and untrained (red) patterns. The time at which the false-negative "miss" rate climbs to 1% (at a fixed 1% false negative rate) is called the capacity (analogous to Fig 1). (**b**) Histogram of synapse ages within a dendrite. Red line shows exponential decay. Synapses reach the end of the age queue at 10–12 learning events in this example. (**c-d**). Capacity graphs comparable to those produced by the analytical model in Fig 4a and 4b. (**e**). Synapse usage and dendrite usage during the storing of one pattern, as a function of dendrite size. Plots are linked by color to overlying capacity plots. (**f**). Capacity for 3 levels of pattern “correlation”, quantified by redundancy factor ρ (solid lines). Peak capacity was still found for dendrites in the range of “a few hundred synapses”. Avoiding duplication of synapses on dendrites almost completely eliminated the deleterious effects of pattern correlations (dashed lines).

Capacity is plotted in Fig 4a as a function of K for pattern density values ranging from 0.8% to 18%. In the case with $f_{A}\;=\;1.5\%$, capacity peaked at ~30,000 patterns when dendrites each contained 256 synapses, and declined substantially for both short (K<100) and long (K>1000) dendrites. As the pattern density increased (to 18%) or decreased (to 0.8%), peak capacity varied nearly 5-fold, favoring sparser patterns, but over the more than 20-fold range of pattern densities tested, peak capacity always occurred for dendrites ranging from 100–500 synapses (grey shaded area). Focusing on the high-capacity (sparse) end of the range with $f_{A}<3\%$, peak capacity was confined to the narrower range of 200–500 (i.e. “a few hundred”) synapses. We also observed that sparser patterns led to a preference for longer dendrites, an effect we unpack below using full network simulations. It is important to clarify that the higher recognition capacity seen for sparser patterns does not result from the fact that sparser patterns contain less information, thereby reducing storage costs per pattern (see S1 Text). We also note that in the more realistic conditions modeled in the full network simulations (see below and Fig 5), peak capacity saturates at slightly higher pattern activation densities (around 1.5%) than is predicted by the analytical model, and the optimal pattern density may be higher still under conditions of increased background noise (S1 Fig shows strong susceptibility to background noise even at 3% pattern density).

To test the effect of pattern noise on capacity, we varied the input noise level by choosing combinations of $N_{burst}$ and $P_{burst}$ whose product was always $\mu_{burst}\;=\;4$ spikes, but that yielded narrow or broad spike count distributions for each active pattern component (Fig 4b, see histogram insets). In this way, we varied the degree to which a trained pattern resembled itself upon repeated presentations. The variation in event counts arising from the above scheme could be viewed as representing either variation in the number of action potentials arriving at the presynaptic terminal from trial to trial, or variation in the number of synaptic release events caused by a given number of action potentials, or a combination of both effects. As expected, higher noise levels reduced peak capacity (Fig 4b), except in the long dendrite range (K>1000) where central limit effects rendered dendrites insensitive to this type of noise. In keeping with this effect, optimal dendrite size increased slightly as the noise level increased, but again, peak capacity was consistently seen for dendrites in the “few hundred” synapse range. Even higher levels of noise were not considered because a simple, biologically available saturation strategy that maps multiple release events into a relatively constant post-synaptic response can largely mitigate the effects of this type of noise. (We did not include a multi-input saturation mechanism in our model to avoid the added complexity).

### Optimal dendrite size depends little on network size

To verify that the preference for dendrites in the few hundred synapse range was not an artifact of “small” network size, we generated capacity curves from Eq 2 for networks scaled up 256-fold from a base size of N = 5.12 million synapses to ~1.3 billion synapses. The results are shown on a log plot in Fig 4c. As shown in Fig 4d, the scaling power for dendrite sizes K = 64, 256, and 1024 were, respectively, 0.98, 0.97, and 0.97, confirming earlier observations that storage capacity in an optimized dendrite-based memory grows essentially linearly with network size [7]. All the while, the preference for dendrites containing a few hundred synapses remained essentially invariant.

### Validating the analytical model with full network simulations

To cross-check the results of the analytical model, we simulated a full memory network, and measured capacity empirically as a function of K. Unlike the analytical case, in which capacity was assumed to be proportional to the calculated length of dendritic age queues, in the network simulations we performed explicit old-new recognition memory tests, and optimized system parameters to achieve false positive and false negative error rates of 1%. In the interests of greater biological realism, we replaced the hard dendritic firing threshold and binary input-output function with a continuous sigmoidal input-output function given by $\frac{1}{1+e^{-s\left(x-\theta_{F}\right)}}$, and optimized over the slope parameter s along with the 4 threshold parameters. In addition, we relaxed the strict assumption of the analytical model that every input to the network was statistically independent of every other, and instead arranged for each input axon to form ρ synaptic contacts within the memory area, rather than just one. This “redundancy” factor, ρ, set by default to 200, introduced some degree of correlation in the input patterns, and lowered peak capacity somewhat, but had no effect on our main conclusions.

Fig 5a depicts one such simulation with 5.12 million synapses. In the top panel, blue dots show responses to trained patterns, red dots show responses to randomly drawn (untrained) patterns that establish the baseline trace strength (green dashed line) above which stored pattern traces must rise to be recognized. Consistent with the analytical model, responses to trained patterns remain essentially constant during an extended post-training period, in this example spanning ~10,000 patterns. After the flat post-training phase, in contrast to the relatively abrupt fall in trace strength envisioned by the analytical model, a more gradual decline is seen, reflecting the variable times at which the synapses encoding each pattern reach the end of their age queues in different dendrites. Note that the false negative error rate begins to climb during this trace decay period, as the lower fringe of the trained response distribution (blue) progressively merges with the untrained background distribution (red). In this simulation, capacity was reached at ~21,000 patterns, which by our specification is the point where both false positive and false negative error rates equaled 1%. Mirroring the approach taken with the analytical model, multiple simulations were run with varying firing, learning, and recognition thresholds to find the combination of parameters that maximized capacity for each value of K, subject to the same error rate constraints as before. As an additional check of the analytical model, we histogrammed synapse ages within a dendrite (for many dendrites) (Fig 5b), and found that they conformed to a geometric distribution as predicted (red line shows a fitted exponential decay), up to the “cliff” at the end of the age queue (blue dashed line).

Capacity was measured for dendrite sizes between 32 and 4,096 synapses, and the results are shown in Fig 5c and 5d, which are the analogues of Fig 4a and 4b, respectively. When compared to the curves produced by the analytical model, the capacity curves produced by full network simulations had similarly placed capacity peaks and similar qualitative dependence on pattern density and noise levels. In one minor difference, we noted that under the more realistic conditions modeled in the full network simulations, peak capacity saturated at slightly higher pattern activation densities (around 1.5%) than was predicted by the analytical model (Fig 4a).

To determine whether the predictions regarding optimal dendrite size would survive under even more challenging “real world” operating conditions, we added increasing amounts of background noise (spurious spikes added to nominally inactive pattern components), on top of the pre-existing burst noise and pattern correlations. As in the case of burst noise, the background noise level varied between 2 extremes: zero noise, which maximized capacity, and a “high noise” level that reduced storage capacity by roughly a factor of 2 compared to the no-noise case. As in the case of burst noise, we did not consider very high noise levels on the grounds that the deleterious effects of background noise can be compensated by a relatively simple mechanism, for which there is evidence: pre-synaptic terminals with low release probability for “singleton” spikes, along with paired pulse facilitation [89], would allow the effects of sporadic background spikes to be suppressed while maintaining strong responses to signal-carrying bursts. Even at background noise levels capable of causing a significant reduction in peak capacity, the effect of background noise on optimal dendrite size was negligible (S1 Fig). Only at very high levels of background noise, where capacity was reduced more than twofold, did optimal dendrite size change significantly, moving outside of the of the “few hundred” synapses per dendrite range (S1 Fig).

Next we examined the effect of increasing correlations in the input patterns. Given that a single axon can in fact form many thousands of synaptic contacts, corresponding to a much higher redundancy factor than we used in our base simulation, we ran simulations using redundancy factors ρ=5,000 and ρ=10,000 (Fig 5f), which meant that groups of 5,000 or 10,000 synapses scattered across the memory were activated identically. Given previous reports that input correlations can be very deleterious to capacity [10], we speculated that these drastic reductions in the effective dimensionality of the input patterns would severely challenge a memory architecture that was designed to perform optimally with random inputs, or at least significantly alter its behavior. As shown in Fig 5f, however, even in the high-redundancy case (with a 10,000-fold reduction in input space dimensionality), peak capacity dropped by only a factor of ~2 compared to the case with ρ=200, with little to no change in optimal dendrite size.

We next took advantage of the full network simulations to probe the mechanisms that lead to the capacity costs associated with both short and long dendrites. Fig 5e shows two important quantities: the average number of dendrites ($\mu_{LD}$) and synapses ($\mu_{LS}$) used to store a single pattern in the simulations from Fig 5c. The significance of these quantities is discussed below as we work through the distinct capacity penalties for long and short dendrites.

### Penalty for long dendrites

**Fig 6:**
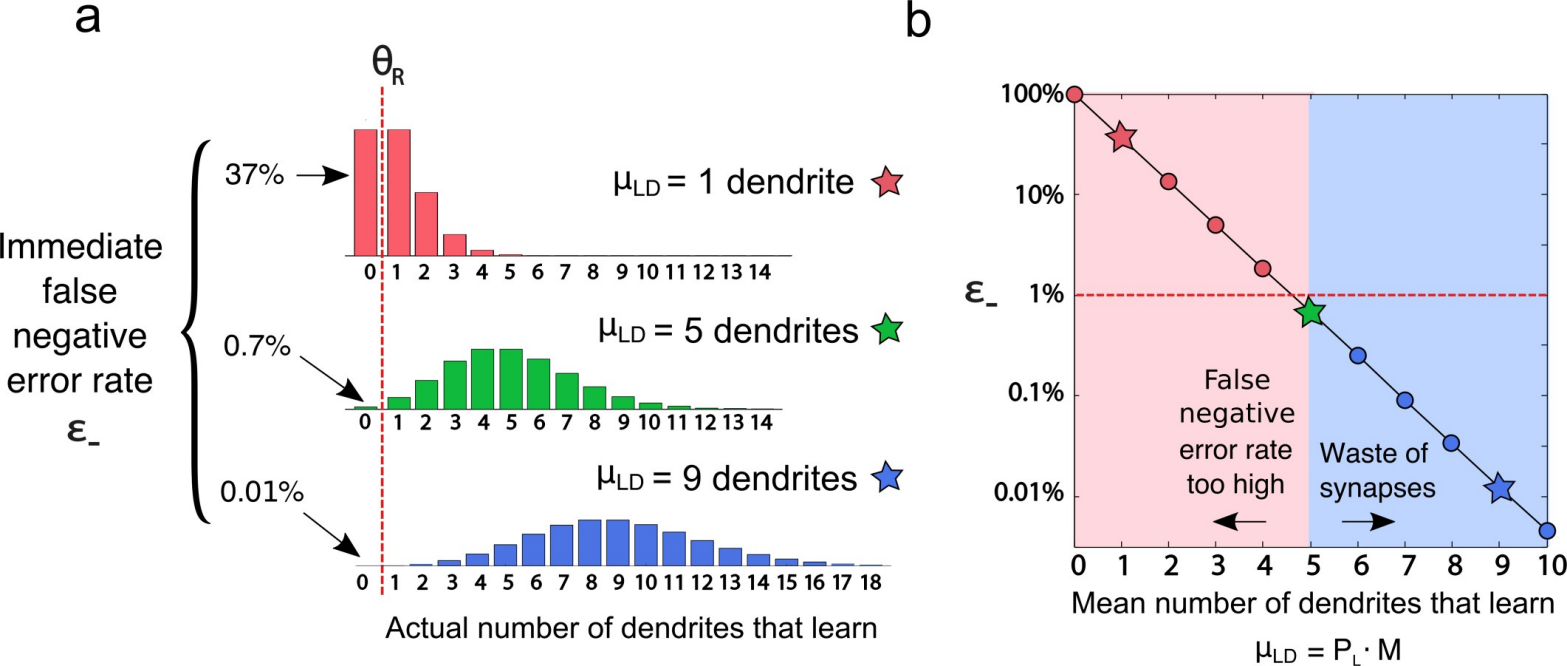
Why a recognition memory of this type must learn in at least 5 dendrites on average to store each pattern. (**a**) Poisson distributions of actual numbers of dendrites that learn for a range of average rates, assuming no spiking noise. Fraction of cases where no dendrites learn establishes the immediate false-negative rate (again assuming no noise). The case with an average usage of 5 dendrites leads to an false negative (FN) rate of 1% immediately after storage. Including input burst noise pushes the optimized dendrite usage slightly higher to ~7 dendrites for a 1% FN error rate (see main text and SI). (**b**) False negative (FN) error rates as a function of average dendrite usage rate. Three stars represented cases from (a).

As shown in Fig 5e, as dendrites grow longer, dendrite usage per stored pattern drops from a value around 10 (at peak capacity) to a “floor” of roughly ~7 dendrites at the long-dendrite end of the range, whereas synapse usage climbs steeply from a baseline of around 150 synapses. To understand the source of the lower bound of ~7 on the average number of dendrites used to store each pattern, it is useful to consider the situation that holds when, in the interests of resource efficiency, we attempt to store each pattern with the minimum possible trace strength: one dendrite. One dendrite firing in response to a familiar pattern is in principle sufficient for recognition, if it is reliable (i.e. occurs > 99% of the time), and if the network’s response to untrained patterns is reliably zero (i.e. > 99% of the time). In a large network, given that each dendrite participates in learning with equal (small) probability, the distribution of the number of dendrites that undergoes a learning event is approximately Poisson with mean $\mu_{LD}\;=\;P_{L\;}\cdot M$. Given that a Poisson distribution is characterized fully by its mean, setting $\mu_{LD}\;=\;1$ by adjusting the learning thresholds, which control $P_{L\;}$, means that one dendrite will undergo a learning event for each presented pattern–on average–which is the goal. However, with a mean of 1, the probability that *zero* dendrites learn is surprisingly high: ~37% (Fig 6a, top plot). Thus, in aiming to use a single dendrite to encode a pattern on average, more than a third of all patterns presented to the network *would produce no memory trace at all*, leading to a false negative error rate far above the 1% acceptable threshold. To avoid this pitfall, it is critical to reduce the probability to below 1% that zero dendrites learn, which according to the Poisson distribution requires a mean $\mu_{LD}\;=\;5$ dendrites. This requires a remarkable 5-fold increase in $P_{L\;}$ relative to the theoretical minimum, with a corresponding 5x increase in synapse resource consumption (Fig 6a, middle plot). Worse, given increased variability in the number of learning dendrites as well as increased readout failures due to input noise and correlations, storage capacity turns out to be maximized when an even higher value of $P_{L\;}$is used, achieved by further loosening the learning thresholds, which for our combination of system parameters leads to the empirically obtained optimal value of $\mu_{LD}\;=\;\sim 7$ dendrites at the long-dendrite end of the range. Given this floor of ~7 dendrites, it becomes clear why synapse usage increases as dendrites grow longer: the number of synapses used in a dendrite that undergoes a learning event is roughly proportional to the dendrite length K, since the number of synapses that learn is roughly proportional to the number of synapses activated in the dendrite, which is proportional to dendrite size. Tied to this increase in synapse usage per pattern, as the total number of dendrites M in the system decreases (because each one contains a larger fraction of the synapses), the frequency with which each dendrite must participate in learning increases, which speeds the per-pattern rate at which synapses move along their age queues. Thus, from a capacity standpoint, it is ideal to choose system parameters such that the minimum encoding bound of 7 dendrites is actually used (or whatever minimum number of dendrites is needed, given the settings of the error rate thresholds and noise level), but having met this lower bound, dendrites should be kept as short as possible.

### Penalty for short dendrites

The reasons capacity declines as dendrites grow shorter are complex, and are discussed only briefly here (see the S1 Text and S3 and S4 Figs for more details). We first consider why dendrite usage increases for short dendrites, rather than remaining at the minimal encoding bound. Short dendrites are intrinsically more susceptible to variability in crossing their learning and firing thresholds, since fewer active synapses are involved. As dendrites become very short, this requires the network to increase dendrite usage far above the nominal lower bound of $\mu_{LD}\;=\;5$. For example, under sparse activation ($f_{A}\;=\;1\%$), medium noise conditions (${P_{burst}\;=\;\frac{4}{7},N}_{burst}\;=\;7)$ with dendrites containing ~200 synapses, when the system is optimized for capacity, $\mu_{LD}\approx 15$ (blue solid curve in Fig 5e), substantially more than the number of dendrites used under maximum capacity conditions. While this increase in dendrite usage is more than offset by the reduced dendrite size, which tends to reduce synapse usage, the total number of synapses altered during learning in fact remains approximately constant, implying that a larger fraction of synapses is modified within each short dendrite that engages in learning. This higher synapse burn rate in short dendrites leads to shorter age queues, and in the end lowers capacity.

## Discussion

The memory architecture we have studied is ordinary, in the sense that it consists of axons making contacts directly onto the neurons whose firing represents the memory trace, but is out-of-the-ordinary among online learning models in that it includes a layer of thresholded dendritic units interposed between the input axons and the final common output of the network.

The main contributions of this paper are (1) Eq 2, which captures the interactions between key variables that influence storage capacity in a dendrite-based online recognition memory, and (2) our showing that over a wide range of input pattern statistics and network sizes, memory capacity is maximized when dendrites contain a few hundred synapses, which corresponds to the typical dendrite size found in medial temporal lobe memory areas [90]. To our knowledge, ours is the first theory that accounts for dendrite size in terms of its role in optimizing online learning capacity.

Beyond the uses we have shown here, our model could potentially be used (1) to help explain why different combinations of parameter settings co-occur in different recognition memory-related brain areas, for example in different animal species whose brains may be larger or smaller, whose sensory codes may be sparser or denser, or whose error tolerances may be tighter or looser; (2) to help distinguish brain areas involved in online familiarity-based recognition memory, the task we study here, from areas such as the hippocampus that (also) contribute to explicit recall [87,88]; and (3) to help identify which changes (e.g., spine loss, dendrite retraction, hyperexcitability, etc.) that occur in neurological disorders, aging and stress, are most directly responsible for producing memory deficits–knowledge that may eventually aid in the design of clinical interventions for those suffering from memory loss.

### Why mid-sized dendrites are optimal for recognition memory

Why are dendrites of “medium” size optimal for storage capacity in the context of an online familiarity-based recognition memory? The simplest explanation is that short dendrites suffer from one set of disadvantages, and long dendrites suffer from another, leaving the optimal dendrite size somewhere in the middle. Short dendrites have relatively noisier post-synaptic response distributions because fewer synapses contribute to the response. As a result, a larger fraction of the synapses on a short dendrite must be modified during learning to ensure that the dendrite's response to previously trained patterns remains comfortably at the upper tail of the untrained pattern response distribution. Increasing the fraction of synapses used within a dendrite during each learning event shortens the dendrite's age queue, which comes at a capacity cost. This effect leads to a preference for longer dendrites.

But long dendrites also have their disadvantages. An online recognition memory should aim to store the weakest possible trace of each learned pattern, which in our framework corresponds to learning in a small number of dendrites near the "minimum encoding bound" (corresponding to ~7 dendrites under the conditions used in our study; see Fig 5e). This means that the longer the dendrites become, the more synaptic resources are consumed by each dendrite that learns, since the number of synapses used per dendrite during a learning event is roughly proportional to dendrite size. Clearly from this perspective, it's best to keep dendrites as short as possible.

The compromise between the need to keep dendrites long enough to avoid noise and age queue problems, and short enough to avoid excessive synapse use per learning dendrite, puts the optimal size around a few hundred synapses for biologically reasonable values of pattern activation density and noise. Of course, our assumptions regarding "biologically reasonable" pattern activation densities and noise levels are informed guesses rather than certain knowledge, and are not likely to be universal constants across brain areas, species and operating conditions. It is therefore possible that the natural dendrite sizes found in medial temporal lobe memory areas are determined in part by factors other than capacity optimization according to Eq 2. For example, developmental constraints, energy constraints, space constraints, and combinations thereof, may have been responsible for pushing the actual dendrite size in one direction or another, away from the optimal length as determined by capacity considerations alone. Nonetheless, it is useful to capture basic relationships between biophysical parameters, wiring parameters, input pattern statistics, and capacity, as a starting point for a more complete online memory model.

That mid-sized dendrites optimize capacity can be understood from another perspective. Eq 2 shows capacity is given by the ratio of L, the length of a dendrite's age queue, to $P_{L}$, the probability that a dendrite learns. $P_{L}$, in the denominator, grows larger as dendrites grow in size because the same average number of dendrites is always used to learn, but when dendrites are long, there are fewer of them to choose from. L, in the numerator, grows smaller as dendrites shrink in size because of the higher value of $f_{pot}$ needed to compensate for noise effects. Balancing these two effects, capacity is maximized for dendrites of intermediate size, for which L is not too small, and $P_{L}$ is not too large.

Thus, among the many roles that dendrites may play in the brain, in the context of an online familiarity-based recognition memory, separately thresholded dendrites play the critical role that they downsize the learning units from neuron-sized units (~20,000 synapses) to units containing a few hundred synapses, which are much more numerous, while still containing enough synapses to avoid the capacity costs associated with noise effects and shortened age queues. Simply put, having separately thresholded dendrites provides the memory system with more learning units of a better size. If dendrite-sized learning units were not available, so that it was necessary to construct an online recognition memory from neuron-sized units, storage capacity would be cut by an order of magnitude or more (see Fig 5c).

### Response variability is bad, so response normalization mechanisms are good

A general theme that emerges from our study is the importance of variability control for a recognition memory. The goal of a neural-style online recognition memory is to store a trace of each learned pattern that consumes as few synaptic resources as possible, but that nonetheless allows the network to produce a reliable recognition response on future encounters with a stored pattern. Variability in the magnitude of network responses to either learned or unlearned patterns, such as that produced by burst noise, or low pattern density, complicates this goal in at least two ways. First, increased variability in the responses to unlearned patterns raises the level of background noise, and thus the required minimum encoded signal strength that learned patterns must obtain. This in turn increases the number of synapses that must be devoted to storing each new memory. Second, increased variability in signal strength for learned patterns increases the rate of readout failures (for fixed firing and recognition thresholds). This increase in false negative errors must again be compensated for by increasing memory trace strength for all patterns, which wastefully strengthens patterns whose traces were already well above the recognition threshold.

These effects imply that a brain system devoted to recognition memory is under intense pressure to include response normalization mechanisms, presumably involving local inhibitory circuits [91–99].

It is intriguing to note that if network behavior could be perfectly normalized, so that every pattern is stored by learning in the exact same number of dendrites, e.g. 1 dendrite, then this would represent a 7-fold resource savings, presumably leading to a corresponding boost in capacity compared to the peak capacity conditions shown in Fig 5 (where an optimized high capacity network chooses to learn using 7 dendrites).

### Existing experimental results that are consistent with our model

Several of the mechanisms and processes in our dendrite-based learning scheme are consistent with known biological mechanisms, including that:

1. Strong stimulation of dendrites can trigger local learning processes, independent of somatic firing [61,65,66,68,74,78–80,86];
2. Under in vivo-like conditions, a local spike in a single dendrite can drive a burst of action potentials at the soma [100];
3. Dendrites have dissociable learning and firing thresholds, ordered such that strong stimulation of the dendrite can trigger LTP, while remaining below the local dendritic firing threshold [86].
4. Individual synapses transition between two (strong and weak) stable states [68,101–105];
5. LTP and LTD occur hand in hand within the same dendritic compartment when a learning event has been triggered (in keeping with the synaptic tagging/cross-tagging hypothesis [68,80,106,107];
6. Synaptic depression can be triggered heterosynaptically when a nearby synapse undergoes LTP, suggestive of a competitive, zero-sum mechanism within a dendritic locale [68,78];
7. LTP and LTD, rather than producing long term stable finely-graded weight changes, appear to primarily (and oppositely) affect synapse survival time [105].
8. Memories encoded by LTP have designated lifetimes, at the end of which they are erased by an active synaptic weakening process involving removal of GluA2/AMPARs [108–110]. Furthermore, blocking this depression process increases memory persistence (108).

### A weak prediction: The compound learning threshold

The main speculative/predictive features of our model pertain to the specific conditions for LTP and LTD. First, following [7] we assumed here that the triggering of a learning event in a dendrite, which induces both LTP and LTD, depends on a compound threshold: in order to learn, a dendrite must both (1) receive an unusually strong presynaptic input, that is, unusually many axons impinging on the dendrite must be firing and releasing glutamate; and (2) experience an unusually strong post-synaptic response, that is, unusually many of the firing axons must be driving synapses that are already in a strong state. Note that a traditional Hebbian learning rule would tie learning to the post-synaptic response alone ($\sum w_{i}x_{i}$), placing no explicit condition on the number of axons participating ($\sum x_{i}$). The pre-synaptic condition was incorporated into our model opportunistically, when we observed that doing so doubled the memory's storage capacity [7]. We call the existence of a compound learning threshold a "prediction" of our model on the grounds that the brain would have been under evolutionary pressure to discover any small functional modifications that significantly boost storage capacity, and so the brain might have “discovered” this optimization–as we did. The prediction is weak, however, given that the memory can function in basically the same fashion with a single, conventional post-synaptic threshold, albeit with reduced capacity.

### A strong prediction: Synapses should have age counters

Unlike our weak prediction of a compound dendritic learning threshold, which could be falsified without dire consequences for the model, the prediction that synapses involved in an online familiarity memory should have a prescribed lifetime in the potentiated state, after which they are actively depotentiated, is a more deeply rooted feature of our model. This prediction is also a nearly inevitable consequence of the statement of the learning problem itself: any online recognition memory whose memory retention is much shorter than the animal's lifetime will be "full" at all times, except for a transient period at the beginning of the animal's life when the memory is first filling up. Once it reaches its chronically full state, each time a new pattern is written into the memory by strengthening synapses, as a matter of homeostatic necessity the equivalent of one stored pattern must be erased by weakening synapses, and in the interests of optimal performance, that one erased pattern should be the oldest stored pattern. The alternative–partially degrading many patterns of varying ages–is a poor strategy for a recognition memory, since any pattern whose signal strength is prematurely degraded to the point where it falls below the recognition threshold is functionally lost, yet its unerased detritus continues to uselessly consume space in the memory. Furthermore, since it is most efficient from a resource allocation point of view to store memory traces that are just strong enough to cross the recognition threshold, and no stronger, the system cannot abide gradual attrition of pattern traces. Thus the problem statement itself, and simple logic, dictate that a memory network in the brain devoted to online familiarity/recognition memory should attempt to target the oldest information for erasure as each new pattern is stored. It is difficult to imagine how selective erasure of old information could occur unless synapses keep track of their ages, and unless a dendrite is able to target its oldest synapses for depression as it undergoes each new learning event.

Age-based depression of synapses was previously explored as a strategy for increasing online learning capacity in the context of a 1-layer Willshaw network [5]. It is only in the context of a 2-layer memory, however, in which synaptic learning probabilities can be driven down to extremely low values without compromising signal strength, that synapses are given the opportunity to actually grow old [7].

### Comparison to online learning models that rely on complex synapses

In the 2-layer dendrite-based memory scheme we have studied, storage capacity is increased (~linearly) by increasing the number of dendrites, without altering the synapse model or the plasticity rule. As an alternative, Stefano Fusi and colleagues have developed two elegant models of online learning that boost capacity instead by increasing the complexity of individual synapses [4,8]. Both models share the following basic framework: the memory consists of N synapses abstracted away from any particular network architecture; by default, every synapse is modified during the storage of every pattern; to store a pattern, synapses are strengthened and weakened in equal numbers; and all instructed weight changes during pattern storage overwrite previously stored information. The goal of these models is to carefully manage the plasticity-stability tradeoff that exists when each synapse is asked to encode information about many patterns that have been stored over time: synapses that are very plastic are good at rapidly storing new information but poor at preserving old information, whereas synapses that are very stable are good at preserving old information but poor at rapidly storing new information (synopsis adapted from [8]).

In the "Cascade" model [4], synaptic weights are binary valued (strong and weak), but can exist in states of varying lability/stability. The state diagram within each synapse operates according to two main principles. First, repeated potentiation instructions push a strong synapse into an increasingly stable strong state, that is, a state that shows an increasing resistance to depression. Similarly, repeated depression instructions by the learning rule have the effect of pushing a weak synapse into an ever more stable weak state, one that increasingly resists potentiation. Second, at "deeper" levels of the cascade, corresponding to more stable strong and weak states, the transitions to even deeper levels corresponding to even more stable states, and the transitions in synaptic weight value from strong to weak or weak to strong, all become increasingly improbable, so that synapses in deeper cascade states remain stable over longer and longer time scales. The variation in these transition probabilities across cascade levels can be considerable: according to [4] the optimal cascade model with 10^6 synapses has 15 cascade levels. With this many levels, the most labile synapses at the top of the cascade change weight with probability 1 (i.e. deterministically) in response to a weight change instruction, whereas the most stable synapses deep in the cascade only change weight with probability 1/16,384 in response to a weight-change instruction. Thus, a weak synapse in its most stable state would need to receive ~10,000 potentiation instructions in a row in order to reach a 50% chance of actually undergoing potentiation.

These two operating principles of the Cascade model are clearly distinguishable from those governing synaptic plasticity in our model. First, in the Cascade model, all synaptic state transitions are probabilistic, whereas in the dendrite-based model, all synaptic state changes are deterministic: during learning, weak synapses receiving the instruction to potentiate do so fully and immediately, and during forgetting, strong synapses that reach the end of their lifetimes are fully and immediately depressed. The logic of synapse durability is also different in the Cascade vs. dendrite-based models. In the Cascade model, when a synapse is first potentiated, it is in its most labile strong state, and therefore most vulnerable to depression. In the dendrite-based model, a synapse that has just been potentiated is in its most durable state, in the sense that it will withstand the largest number of consecutive learning events in which it does not participate before it "ages out" and finally succumbs to synaptic depression.

In the Benna and Fusi model [8], the machinery contained within each synapse consists (metaphorically) of a chain of connected fluid-filled beakers. The first beaker represents the synapse’s (graded) strength value by the level of virtual liquid relative to equilibrium, and the last beaker is tied to the equilibrium liquid level. Synaptic potentiation occurs deterministically, and consists of adding a fixed amount of liquid "weight" to the first beaker; synaptic depression consists of removing that amount of liquid from the first beaker. The equilibration of liquid levels in the beaker chain following an instructed weight change, and particularly the equilibration of the first beaker, captures the time course of the memory decay at each synapse. In the example shown in [8], a synapse consisted of a chain of 12 virtual beakers that doubled in capacity with each step down the chain (so that the last beaker had a capacity 2,048 times that of the first beaker), and whose fluid levels were governed by differential equations with pre-determined rate constants linking each pair of buckets. As a practical matter, the authors found the number of discrete levels per beaker could be reduced linearly from 35 in the first (smallest) beaker, corresponding to 35 levels of visible synaptic weight, down to 2 levels in the last (largest) beaker. This parameterization yielded a total of ~10^14 possible memory states within each synapse. Interestingly, unlike the cascade model whose synapses only change state in response to plasticity instructions (which can occur asynchronously), the chain-of-beakers model, if taken literally, continues to equilibrate—i.e. forget—even during periods when the rate of new learning slows or stops, such as during quiet wakefulness or sleep. Thus, an additional layer of mechanism is presumably needed to modulate the inter-beaker flow rates in a coordinated fashion depending on the external learning rate.

In summary, both of these models [4,8] achieve longer memory lifetimes by increasing the complexity of the synapse model as the size of the memory increases. In terms of cost, the machinery inside these more complex synapses requires more parameters (>10), and those parameters must span large dynamic ranges (>1000) to reach realistic memory sizes.

How does a dendrite-based model grow storage capacity without increasing the complexity of the individual synapses? Within virtually any recognition memory model, the conceptually simplest way to increase storage capacity is to reduce the fraction of synapses that are modified during the storage of each pattern (the signal), while correspondingly reducing the response of the memory to random input patterns (the noise). Practically, this can be achieved by sparsifying the input patterns inversely with pattern size as the memory grows larger. Thus, if the memory increases in size from N to c·N synapses, in order to increase capacity c-fold, the pattern density 'a' must be reduced c-fold so that the same number of synapses is activated by each pattern as before. Assuming the learning rule instructs each activated synapse to become strong if it was weak, a·N/2 weak synapses would be potentiated on average (under the assumption that half of the synapses are strong), and an equal number of strong synapses would be depressed to maintain homeostasis (drawn from the N/2 strong synapses). To a rough approximation, this leads to a capacity of ~1/a patterns. Thus, if a = 1% of synapses are changed during the storage of each pattern, then after ~100 patterns are stored, the memory will have turned over completely. This simple scaling approach runs into the biological plausibility problem that very large capacities require very low pattern densities, and very low depression probabilities. To achieve a capacity of 100,000 patterns, for example, only 1 in 100,000 input neurons could be active, and synaptic depression would occur in only 1 in 100,000 strong synapses. Reliably controlling such small activity and plasticity probabilities could be difficult to achieve in neural tissue.

### Dendrites provide a means for sparsifying plasticity without sparsifying patterns

As an alternative both to this very simple sparsification approach, and to the "complex synapse" approach developed by Fusi and colleagues, adding a layer of dendritic learning units allows the memory to push further into the sparse plasticity regime without the need for very low pattern densities or plasticity probabilities. Relative to a flat (1-layer) memory model, dendritic learning thresholds can restrict learning to just a few dendrites from a very large pool. For example, in a simulation of a 5 million-synapse network discussed previously, with a moderate pattern sparseness level of a = 3%, the dendrite learning probability after optimization was P_L_ = 0.0005, (corresponding to 1–2% of neurons in the network having one dendrite that crosses the learning threshold). Beyond the sparsification of learning attributable to dendritic learning thresholds, learning is sparsified even further by the fact that within each learning dendrite, only the active 3% of synapses receives (and obeys) the instruction to potentiate or refresh, and that same small fraction of synapses is depressed. Thus, in the above scenario, relative to a 1-layer network with the same coding density of 3%, the existence of a dendritic learning threshold sparsifies learning by a factor of 1/P_L_ = 2000, significantly boosting capacity without requiring extreme, biologically unrealistic coding sparseness.

### Regarding the experimental detection of sparse dendritic learning events

In our model the formation of new memories is achieved through long-term potentiation (or rejuvenation) of a few activated synapses on a few strongly activated dendrites that undergo learning events. The "forgetting" of old memories involves heterosynaptic depression of the least-recently-potentiated/rejuvenated synapses in the same dendrites that are undergoing learning. Given the pressure to keep memory traces at their bare minimum strength, when our model is optimized for capacity, synaptic changes are exceedingly sparse, involving only a small fraction of the synapses on a minute fraction of dendrites. (The finding that memory capacity is optimized by sparse patterns has also been reported for 1-layer models: [2,111–114]). For example, in a memory network containing ~5 million synapses, under conditions that optimize storage capacity (i.e. with dendrites containing ~256 synapses, and patterns of 3% density), we found that each time a pattern is learned, only 150 of the 5 million synapses learn (0.003%), less than half of which are overtly strengthened (i.e. some are only rejuvenated), and those few altered synapses are confined to just 10 of the 20,000 dendrites contained within the network. If we consider extremely sparse synaptic plasticity to be a prediction of our model, could such sparse changes be detected experimentally? The likelihood of detecting changes in this few dendrites seems higher when it is considered that 20,000 dendrites corresponds to 500–1,000 neurons. We would thus expect that 10 (i.e. 1–2%) of the neurons in the network would contain a dendrite that participates in learning. In vivo imaging techniques with a field of view containing hundreds of neurons should make this level of detection possible.

### What is the role of structural plasticity in online learning?

What role might structural plasticity play in online learning? We previously explored the role that active dendrites might play in familiarity-based recognition in the very different scenario where patterns can be trained repeatedly [46,115]. The opportunity for repeated, interleaved training of patterns gives the system time to exploit wiring plasticity mechanisms [116], wherein existing connections between axons and dendrites can be eliminated and new ones formed in such a way that correlated inputs end up forming contacts onto the same dendrites. This type of wiring plasticity is not an option in an online learning scenario, since each pattern is experienced only once, such that all learning-related synaptic changes must be immediate–or at least immediately induced. We showed that correlation-based sorting of inputs onto different dendrites using a Hebb-type learning rule increased the storage capacity of a neuron by more than an order of magnitude compared to a neuron with the same total number of synaptic inputs that lacked dendrites. Furthermore, as here, we found that dendrites of intermediate size optimized capacity–though for different reasons.

It is interesting to note that in our current model, structural turnover of weak synapses has no effect on what is stored in the memory, as long as new weak synapses are added to the system at the same rate that existing weak synapses are removed. If weak synapses form a substantial fraction of the total synapse population–we have assumed 50% here (but the percentage may actually be closer to 90% in CA1 –see [117])–then high rates of spine elimination and new spine formation could be tolerated within the memory area without any loss of stored information–again, as long as the turnover is restricted to weak synapses. What would be the advantage of eliminating existing weak connections and forming new ones? Under the assumption that input axons are uncorrelated, as we have assumed in this work for simplicity, we can see no advantage to this type of structural turnover. However, if meaningful correlations between input axons do exist, then structural turnover could be a sign that wiring plasticity mechanisms are attempting to co-locate correlated synapses on the same dendrites [118,119], which *could* lead to a significant capacity advantage [46,115,116].

### Relationship to other forms of memory

Familiarity-based recognition is a very basic form of memory, and is most closely associated with the perirhinal cortex [10,87,88]. However, currently available data regarding the responses of familiarity (vs. novelty) neurons in the PRC is complex, and not easily related to our findings here (see S1 Text for an in depth discussion). Further work will be required to determine whether the dendrite-based architecture of Fig 2b will be helpful in explaining familiarity-based recognition processes in the brain.

What can the dendrite-based architecture we have studied here tell us about other types of memory systems? A trivial extension of our architecture in which N copies of the memory network are concatenated would allow the construction of a full N-bit binary online associative memory. This type of memory would behave exactly as ours, but would allow an arbitrary N-bit output pattern to be one-shot associated with each input pattern, as in a Willshaw network. In this scenario, only the subset of the N networks whose outputs are instructed to be 1 would learn each input pattern, while any networks instructed to produce 0 responses would simply ignore the input pattern. If the output patterns are sparse (which they needn't be), only a small fraction of the networks would need to participate in the learning of each association.

It might also be desirable to assign extended lifetimes to particularly important patterns; this could be accomplished in either of two ways: 1) Extended-lifetime synapses could be established during the learning of important patterns, so that the synapses representing those patterns would remain invulnerable to depotentiation for longer times, or even permanently. Doing so would of course reduce the lifetimes of other patterns in the memory. 2) The memory could be composed of multiple subnetworks having a range of pattern lifetimes, and important patterns could be stored in longer-lifetime (i.e. larger capacity or more rarely used) networks. The decision as to which or how many networks participate in the storage of each pattern could be gated by an "importance" signal provided by another brain area.

In other cases it might be valuable to store different trace strengths for different patterns, rather than uniform, bare-bones recognition traces for all patterns. Note this goal is inconsistent with the goal to maximize storage lifetimes for all patterns, but could also be useful in certain ecological situations. Our simple architecture allows for this directly: nothing is to prevent a larger or small number of dendrites from being used in the learning of any particular pattern, such that it's memory trace would be stronger or weaker than the norm. Regardless of trace strength, a pattern’s lifetime would remain roughly the same, since lifetimes are determined mainly by the lengths of the dendritic age queues, which do not depend on the number of dendrites used for storage. The trace strength assigned to each pattern could again be determined by a signal generated by another brain area, whose effect is to raise or lower dendritic learning thresholds.

In yet another scenario it might be useful to store gradually decaying memory traces so that trace strength can represent recency of learning (which is again a different goal than maximizing recognition capacity). A graded recency signal can be efficiently produced by storing each pattern simultaneously in multiple networks with a range of capacities/sizes/memory lifetimes. Early in its storage lifetime, the pattern would evoke a memory trace from all networks, so that it's total trace strength would be high, but as time progresses, and its trace progressively expires from the lower-capacity networks, its overall trace strength would gradually decay. This use of such a tiered system to achieve a graded decay time course is more resource-efficient than certain other forms of trace decay that have been considered in the online memory literature, in that the stored information in a tiered network with synapse age management expires in a controlled fashion [109].

Finally, it will require future work to determine which of our results can carry over to Hopfield-style recurrent networks [120–123] constructed from neurons with thresholded dendrites, where the goal in that case would be to maximize recall capacity. In one obvious difference, the ability to recall entire patterns from partial cues requires that the entire patterns be stored (in stark contrast to the need to generate only a reliable familiarity signal), so synapse resource consumption per pattern will be much higher than in the basic familiarity network. Furthermore, the need to modify recurrent synapses during the initial learning of a pattern implies that the participating neurons must fire action potentials during initial learning in order to activate those recurrent connections, which implies that their dendrites must cross both the learning and firing thresholds during learning. Interestingly, this requirement would seem to render such a memory useless for familiarity-based recognition, since the neurons that participate in the learning of a pattern must already fire on a pattern’s first presentation to the memory. This incompatibility could be one reason why the functions of familiarity and recall memory have been assigned to distinct areas within the medial temporal lobe [87,88].

## Methods

### Notation

$\alpha_{ij}$ Age (in number of learning events) of synapses connecting axon *i* to dendrite *j*
$a_{pre}^{\left(j\right)}$ Pre-synaptic activation of dendrite *j*
$a_{post}^{\left(j\right)}$ Post-synaptic activation of dendrite *j*
*Bi(n*,*p)* Binomial distribution function with *n* trials and success probability *p*
*C* Memory capacity of network, measured in number of patterns
$D_{j}$ Set of inputs connected to dendrite *j*
ϵ_*±*_ Error rates (plus for false positive, minus for false negative)
*f*_*A*_ Pattern activation density (i.e. fraction of axons active in a given pattern)
*f*_*pot*_ Average fraction of synapses that learn (i.e. are potentiated or juvenated) within a dendrite during a learning event ($f_{pot}\;=\;\frac{\theta_{Lpre}}{K}$)
*f*_*age*_ Average fraction of strong synapses in a dendrite that age during a learning event
*f*_*S*_ Fraction of synapses in a dendrite that are strong (equal to 50% in our networks)
*K* Number of synapses per dendrite ($K\;=\;N_{S}/M$)
*L* Length of the age queue, measured in number of learning events
*M* Number of dendrites in the network ($M\;=\;N_{S}/K$)
*N*_*A*_ Number of axons providing inputs to the network, defining the dimensionality of the input
*N*_*burst*_ Number of trials used in generating synaptic burst noise from a binomial distribution
*N*_*S*_ Total number of synapses in network ($N_{S}\;=\;M\cdot K$)
*P*_*burst*_ Probability used in generating synaptic burst noise from a binomial distribution
*P*_*F*_ Probability that a random dendrite fires upon presentation of a random untrained pattern
*P*_*L*_ Probability that a random dendrite is involved in the learning of a random pattern
*r*_*j*_ Binary output of the *j*^*th*^ dendrite (signifying whether the dendrite fired or not)
*r* Output of the memory network measured in the number of dendrites that fired
*s* Slope parameter for dendritic activation sigmoid (only used in simulations)
*θ*_*±*_ Maximum tolerated error rate (plus for false positive and minus for false negative)
*θ*_*F*_ Firing threshold for a dendrite (in spikes)
*θ*_*Lpost*_ Post-synaptic learning threshold (in spikes arriving at strong synapses)
*θ*_*Lpre*_ Pre-synaptic learning threshold (in spikes arriving at strong or weak synapses)
*θ*_*R*_ Recognition threshold for network to distinguish familiar from novel patterns (in number of dendrites)
*µ*_*burst*_ Mean number of spikes produced in a burst by an active synapse ($\mu_{burst}\;=\;N_{burst}\cdot P_{burst}$)
*µ*_*LD*_ Average number of dendrites used for learning one pattern
*µ*_*LS*_ Average total number of synapses used for learning one pattern
*µ*_*pre*_ Average presynaptic activation for a random pattern
*µ*_*pot*_ Average number of synapses per dendrite used for learning one pattern
*w*_*ij*_ Weight of synaptic connection from axon i to dendrite j
x Sparse, binary-valued vector representing an input pattern
$\tilde{x}$ Sparse, random, integer-valued vector representing the number of spikes arriving at each synapse

### Calculating memory capacity

As discussed in the main text, after a certain number of learning events has occurred following the storage of a pattern feature in a dendrite, the strong synapses encoding the stored feature begin to “fall off” the end of the dendrite’s age queue, and the memory trace in the dendrite is effectively lost. We refer to the number of learning events that can be endured before this loss occurs as the length of the age queue *L*. If we assume that the frequency of learning events is constant across dendrites in the network, given that the queue length *L* is also constant across dendrites, most of the strong synapses encoding a particular pattern’s features will be depressed roughly simultaneously (in different dendrites), leading to a relatively rapid decay of the network’s overall response r to that pattern. The value of L is therefore a measure of the length of time that a pattern’s trace persists in the memory, and is therefore effectively a measure of capacity in units of dendritic learning events.

L can, in principle, be determined by framing learning as a Markov process with the state diagram shown in Fig 3. Consider a single synapse on a given dendrite. If $\vec{p}$ is the (L+1)×1 vector containing the probability that, at a given time, this synapse is in each of the L+1 states shown in Fig 3, and T is the $\left(L+1\right)\times \left(L+1\right)$ matrix containing the state transition probabilities, then with each learning event, $\vec{p}$ will change as $\vec{p}\;\to T\vec{p}$. After many learning events, $\vec{p}$ will approach the equilibrium distribution, characterized by the condition that learning leaves it unchanged: ${\vec{p}}^{\infty }\;=\;T{\vec{p}}^{\infty }$. Using the fact that for the equilibrium distribution ${\vec{p}}^{\infty },\;f_{s}$ of the synapses must be strong, one can solve for L (since the (L+1)×1 vector ${\vec{p}}^{\infty }$ implicitly depends on L). Using the eigenvectors and eigenvalues of T, one can also compute the distribution $\vec{p}\left(t\right)$ after any number of learning events. However, while the Markov approach is very general, the simple dynamics of the age queue allow for a more direct and transparent derivation of L.

To find L, we might naively divide the total number of strong synapses per dendrite ($f_{S}∙K$) by the average number of synapses potentiated in each dendrite that experiences a learning event $\mu_{pot}$. where $\mu_{pot}\approx \frac{\theta_{Lpre}}{\mu_{burst}}$. In words, $\mu_{pot}$ is approximately equal to the total number of spikes impinging on all activated synapses on the dendrite, given by the threshold value $\theta_{Lpre}$ (since in most cases learning dendrites will have just crossed this threshold), divided by the average number of spikes per participating synapse $\mu_{burst}$. This gives $L\approx \frac{f_{S}∙K∙\mu_{burst}}{\theta_{Lpre}}$. However, this would underestimate *L* because synapses that are only juvenated (i.e. that were already strong) do not contribute to the aging of synapses further along the age queue, so that the average rate of progression along the age queue slows as strong synapses grow older. To estimate L more accurately, consider the equilibrium distribution of synapse ages in the queue of a single dendrite (blue histogram in Fig 3). The age of the right-most column of the age histogram is an indicator of the expected age (measured in learning events) at which the synapses encoding a pattern are depressed and moved to the unordered collection of weak synapses. During each learning event, a large fraction ($f_{age}$) of synapses in each column that were *not* activated move rightward to the next older column, while a small fraction ($1-f_{age})$ are juvenated (promoted to the first column). This process leads to a bias towards younger synapses in the queue, and can be well-approximated by a finite geometric sequence with length L, decay ratio $f_{age}$, sum $f_{S}∙K$ (note the sum of the columns is the total number of strong synapses), and first column height $\mu_{pot}$ (the average number of synapses that learn per dendrite per learning event), so that:

$$f_{S}\cdot K=\mu_{pot}\cdot \frac{1-f_{age}^{L+1}}{1-f_{age}}.$$

Assuming that the synapses in a dendrite are all equally likely to be potentiated (ignoring the effects of the postsynaptic threshold–see below), with $\mu_{pot}\approx \frac{\theta_{Lpre}}{\mu_{burst}}$, then we have that $f_{age}\approx 1-\frac{\theta_{Lpre}}{K∙\mu_{burst}}$ and can solve the above equation for L. Note that L counts the number of dendritic learning events before a memory is eroded, whereas memory capacity *C* should count the number of training patterns. Thus, to approximate C, we must multiply L by the approximate number of patterns per dendritic learning event, or “learning interval” $\frac{1}{P_{L}}$, where $P_{L}$ is the probability that an arbitrary dendrite learns a particular pattern. Although $P_{L}$ is conceptually simple, its expression is complicated since it depends on pattern density, noise level, two learning thresholds, dendrite size, and $f_{S}$ (see expression below). Collecting these results, we can approximate memory capacity by

$$C\approx \frac{L}{P_{L}}=\frac{1}{P_{L}}\cdot \left[\frac{\log(1-f_{S})}{\log\left(1-\frac{\theta_{Lpre}}{K\cdot \mu_{burst}}\right)}-1\right].$$

For simplicity, the expression for L in the capacity equation does not include the effect of the postsynaptic threshold $\theta_{Lpost}$, which makes strong synapses more likely to learn, lowers $f_{age}$ and increases absolute capacity. The synapse age distribution remains roughly geometric, however (see Fig 5b), and we observed that the qualitative behavior of the system depends only weakly on $\theta_{Lpost}$, justifying its omission from the analysis.

### Derivation of $P_{L}$

Synaptic activation on a dendrite is governed by 4 binomial random variables: $a_{s}$, the number of active strong synapses; $s_{s}$, the number of spikes received by strong synapses; $a_{w}$, the number of active weak synapses; and $s_{w}$, the number of spikes received by weak synapses. These random variables have the distributions shown below. Learning occurs when presynaptic activation crosses the presynaptic learning threshold, or $s_{s}+\;s_{w}>\theta_{Lpre}$, and postsynaptic activation crosses the postsynaptic learning threshold, or $s_{s}>\theta_{Lpost}.$ Using the distributions for $a_{s},a_{w\;},\;s_{s},$ and $s_{w}$, and the fact that $P_{L}\;=\;p\left(s_{s}+\;s_{w}>\theta_{Lpre},\;s_{s}>\theta_{Lpost}\right),\;$we can write an explicit expression for $P_{L}$:

$$a_{s}\;\sim \;Bi\left(f_{s}\cdot K,f_{A}\right)$$

$$s_{s}\;\sim \;Bi\left(N_{burst}\cdot a_{s},p_{burst}\right)$$

$$a_{w}\;\sim \;Bi\left(\left(1-f_{s}\right)K,f_{A}\right)$$

$$s_{w}\;\sim \;Bi\left(N_{burst}\cdot a_{w},p_{burst}\right)$$

$$P_{L}=\underset{\begin{array}{c} i\in \left[0,f_{s}\cdot K\right]\\ j\in \left[0,\left(1-f_{s}\cdot K\right)\right]\\ k\in \left[\theta_{Lpost}+1,N_{burst}\cdot i\right]\\ l\in [\theta_{Lpre}-k+1,N_{burst}\cdot j] \end{array}}{\sum }Bi\left(N_{burst}\cdot j,p_{burst}\right)\left[l\right]\cdot Bi\left(N_{burst}\cdot i,\;p_{burst}\right)\left[k\right]\cdot Bi\left(f_{s}\cdot K,f_{A}\right)\left[i\right]\cdot Bi\left(\left(1-f_{s}\right)K,f_{A}\right)\left[j\right]$$

where $Bi\left(N,p\right)[k]$ is the binomial pdf with parameters (N,p) evaluated at k. A simpler alternative to evaluating this expression directly is to estimate it by generating a large number of samples of $a_{s},a_{w\;},\;s_{s},$ and $s_{w}$ according to the above distributions, and directly observing the fraction of cases that cross both learning thresholds .

### Checking error tolerances

Once the capacity formula is used to calculate *how long* a given memory trace will last, we must verify that during its lifetime, the trace is sufficiently *strong*. We do this by checking whether the error tolerances $\epsilon_{+}$ and $\epsilon_{-}$ are met immediately after training.

First, we compute $\epsilon_{+}$, the probability that an untrained pattern will be recognized. To be recognized, a pattern must activate at least $\theta_{R}$ dendrites in the network. For a randomly selected untrained pattern, the distribution of the number of activated dendrites will be approximately Poisson with mean $P_{F}\cdot M$, where M is the number of dendrites in the network and $P_{F}$ is the probability that a given dendrite fires in response to a randomly selected pattern. For a pattern to fire a dendrite, it must cause a postsynaptic activation $>\theta_{F}$, or $s_{s}>\theta_{F}$, using the notation of above. Since the distribution of $s_{s}$ is known, it is relatively easy to write an expression for $P_{F}$ and $\epsilon_{+}$ explicitly:

$$P_{F}=p\left(s_{s}>\theta_{F}\right)=\;\underset{\begin{array}{c} i\in \left[0,f_{s}\cdot K\right]\\ j\in \left[\theta_{F},N_{burst}\;\cdot i\right] \end{array}}{\sum }Bi\left(N_{burst}\cdot i,\;p_{burst}\right)\left[k\right]\cdot Bi\left(f_{s}\cdot K,f_{A}\right)\left[i\right]$$

$$\epsilon_{+}=\underset{r\;\geq \;\theta_{R}}{\sum }Poiss\left(P_{F}\cdot M\right)\left[r\right]$$

As for $\epsilon_{-},$ the probability that a previously trained pattern is forgotten, we approximate this quantity with $\epsilon_{-}^{0}$, or the immediately post-training false negative rate (justified by the fact that during the “lifetime” of the memory, C, the trace strength is roughly constant). To calculate $\epsilon_{-}^{0}$, we use the following observation: when training a new pattern, it will learn in a certain set of dendrites. Immediately after training, if the pattern is re-presentated to the network, *all* of these dendrites should respond, since learning has significantly boosted the pattern’s features in these dendrites. In other words, dendrite readout failures immediately after learning should be very rare. Therefore, for a pattern to be too weak for recognition immediately after training, it must have learned in too few dendrites. The number of learning dendrites for a given pattern will have a Poisson distribution with mean $P_{F}\cdot M$. Therefore, $\epsilon_{-}$ can be written

$$\epsilon_{-}\approx \epsilon_{-}^{0}=\;\underset{l<\theta_{R}}{\sum }Poiss(P_{L}\cdot M)\left[l\right]$$

If for the given settings of the learning and firing thresholds $\left(\theta_{Lpre},\;\theta_{Lpost},\;\theta_{F},\;\theta_{R}\right)$, the error tolerances are met–that is, $\epsilon_{+},\;\epsilon_{-}<1\%–$then the memory lifetime is compared to the best memory lifetime found so far. Otherwise, we continue the search through threshold space.

### Code availability

All data contained in figures as well as simulation code is available in S1 Data file titled "Plos data/code".

## Supporting information

S1 Text Additional material discussing effects of various network parameters on memory capacity. (DOCX)

S1 Data Network simulation code and data. (ZIP)

S1 Fig Effect of background noise on network performance. In the base case without background noise, nominally inactive axons (which were the vast majority) never fired. For the medium and high noise cases, nominally inactive axons emitted one spike with the indicated probability. The fraction of inactive axons that fired a spike was chosen so that in the medium case, aberrant spikes totaled approximately 10% of the number of “real” pattern spikes (recall that each active axon generated a burst of 4 spikes on average), and in the high noise case, aberrant spikes were 25% of the real spikes. Increasing background noise decreased memory capacity, and, at high noise levels, pushed the optimal dendrite size to shorter values. For all simulations here, the dendritic activation slope parameter was set to 3.
(TIF)

S2 Fig Network responses for perturbed patterns. The memory network was trained as normal to maximize old/new recognition capacity. We then tested how a trained network responded to perturbed versions of stored patterns. As expected, as an increasing fraction of training pattern bits were changed, network response decreased (black curve). For example, when 20% of an original training pattern’s active bits were assigned to different input lines (keeping pattern density unchanged), average network response fell to roughly one third of the original response. We then tested whether the network could reliably distinguish between exact trained patterns and perturbed patterns (red curve). The network was able to distinguish exact training patterns from 20% perturbed patterns with 85% accuracy.
(TIF)

S3 Fig Explanation of dendrite "availability" problem faced by short dendrites, and the remedy. (See S1 Text for details).
(TIF)

S4 Fig Contributors to additional capacity costs for short dendrites. (**a**) Distributions of pre-synaptic responses to random patterns for dendrites of varying size. (**b**) Same graph as (a) but with responses normalized to the mean response. Colored arrows indicate points where the upper 1% of the probability mass begins, to illustrate that shorter dendrites have larger response variability relative to their mean than longer dendrites. (**c**) Fraction of synapses used within each dendrite involved in learning increases for short dendrites. (**d**) Comparison of capacity for 3 cases with equivalent synapse usage (red dots); capacity drops linearly for shorter dendrites because of the higher values of *f*_*pot*_.
(TIF)
