# Supplementary material for: How Dendrites Affect Online Recognition Memory
Source: PLoS Comput Biol. 2019 May 3;15(5):e1006892. doi: 10.1371/journal.pcbi.1006892 (PMC6527246; doi:10.1371/journal.pcbi.1006892)
Supplement: S1 Text — (DOCX) [file pcbi.1006892.s001.docx]

## SUPPORTING INFORMATION

## The Effect of Background Noise on Memory Capacity and Optimal Dendrite Size

To test whether our conclusions regarding optimal dendrite size depended on the type of noise included in the input patterns, we ran additional simulations that also included “background noise”, in which nominally inactive pattern elements produced spikes with non-zero probability. Just as for foreground burst noise (which was kept at default levels in these runs), background noise was randomly regenerated each time a pattern was encountered, so that patterns “looked different” between training and testing phases. Capacity curves for zero, medium, and high background noise cases are shown in Fig. S1. In the medium-noise case, in which the number of background spikes was set at 10% of the number of pattern spikes, peak capacity was reduced by roughly 20%, with little to no shift in optimal dendrite size. In the high-noise case, where background spikes amounted to 25% the number of pattern spikes, capacity was reduced more than a factor or 2, and optimal dendrite size moved lower, to $K$ = ~100 synapses. Thus, high levels of background noise, if they cannot be avoided, lead to both a significant capacity cost, and a preference for shorter dendrites.

Network Responses to Perturbed Versions of Previously Trained Patterns

Inclusion of noise in stored patterns in effect tests a memory’s ability to “generalize”, since the system is asked to respond similarly to a pattern despite perturbations by noise. Given that *specificity* can also be considered an important quality for a memory, we tested a trained network with novel patterns that were perturbed versions of stored patterns, in lieu of randomly drawn (i.e. completely novel) test patterns as in Fig. 5. As shown in Fig. S2, network responses fell off rapidly as patterns were perturbed from their original trained form. For example, when a test pattern differed from a recently trained pattern in just 20% of its active components, the network response fell by almost a factor of 3 on average, and the perturbed pattern could be discriminated from its original with 85% accuracy (see [1] for a comparison to human one-shot memory performance with perturbed test patterns). Thus, a 2-layer dendrite-based memory architecture, even when trained to discriminate familiar patterns from randomly drawn test patterns, still shows a high degree of specificity for the actually trained patterns. This result (demonstrating specificity) can be reconciled with the previous result (demonstrating generalization) by noting that in the background noise case, system thresholds were optimized to achieve the required 1% error tolerances *in the presence of background noise*, which forced the system to generalize, whereas in the latter case, there was no change during training, only a post-training change in the makeup of the test patterns.

Short Dendrites are Sub-Optimal Due to Dendrite Availability and Reliability Problems and Shortened Age Queues

Given that only ~7 dendrites are needed to encode patterns with high reliability (see section “Penalty for Long Dendrites” in main text), why is capacity not maximized for the shortest possible 7 dendrites? To answer this question, we will first explain why dendrite usage increases for small *K*. Then, we will explain why, nevertheless, synapse usage remains constant. Finally, we will explain why, despite the constant usage of synapses, capacity decreases anyway.

In the main text, we developed the rationale for the minimal encoding strength bound on *µ_LD_*. Why, for small dendrite sizes, does the optimized system not chose to learn with this minimal number of dendrites? To address this question, we will need to examine the probability densities of a dendrite’s presynaptic activation values $a_{pre}$ in response to untrained patterns (Fig. S3, upper plot) and discuss the constraints on the learning and firing thresholds, *θ_Lpre_* and *θ_F_* (*θ_Lpost_* plays no significant role in this discussion and so will be temporarily ignored). First, we note that *θ_Lpre_* must be higher than *θ_F_*, since if it were smaller than *θ_F_*, some dendrites could cross *θ_Lpre_* and undergo a learning event, but then fail to cross *θ_F_* to contribute to readout, thus squandering memory resources. Also, a high *θ_F_* is desirable in order to minimize false positive responses to untrained patterns, while a low *θ_Lpre_* conserves synaptic resources – recall that $\mu_{pot}\approx\theta_{Lpre}$. This combination of constraints tends to pin *θ_Lpre_* just above *θ_F_*, so that we may approximate them as being equal (Fig. S3). However, too high a value of *θ_F_* pushes *θ_Lpre_* so high that there are, on average, not enough dendrites that cross this threshold and are “available” for learning, leading to undesirably small stored memory traces. This situation is depicted in the top plot of Fig. S3 and the subplot for Scenario (1). The relationship between the upper plot and lower two subplots is that the upper plot shows the pre- and post-synaptic response pdfs, while the lower plots show the area under the pdf tails. That is, the green curve in (1) shows *P_F_*, which is equal to the area under the distribution of *a_post_* that is above any particular value of *θ_F_*; the purple curve shows *P_L_*, which is equal to the area under the distribution of *a_pre_* that is above any particular value of *θ_Lpre_*. Note that in the lower plots we have “zoomed in” relative to the upper plot, in order to examine the tiny probabilities (~10^-5^-10^-7^) in which we are interested. On the left side of the two subplots, we have also plotted as bar plots the distributions of the responses to trained and untrained patterns that result from our particular choice of *θ_F_* and *θ_Lpre_*. These are analogous to the histograms from Fig. 6a, but tipped over on their sides. For the excessively high setting of the thresholds depicted in Scenario (1), which leads to an extremely low value of *P_L_*, we see that an insufficient number (<1) dendrites are “available” for learning on average, leading to an unacceptably high rate of false negative errors. To compensate for this availability problem, *θ_F_* must be reduced to allow a lower *θ_Lpre_*, which in turn makes more dendrites available for learning and leads to larger stored memory traces (Scenario 2). However, there is a complication. Lowering *θ_F_* also increases the response to untrained patterns, further increasing the mean number of dendrites *µ_LD_* that must learn in order to maintain recognizable memory traces. Fortunately, *P_L_* rises faster than *P_F_* as both thresholds are decreased together, so the dendrite availability problem can be overcome and the error tolerance satisfied (though at the cost of reduced capacity). Recall, however, that we assumed every encoding dendrite responds with perfect reliability on presentation of a trained pattern (i.e. no readout failures). In fact in the case depicted in Scenario (2), immediate readout failures occur ~30% of the time, requiring that even more dendrites be used to store memories. In particular, in Scenario 2, in order to achieve a mean readout of 14 dendrites, 20 dendrites must learn on average. Thus, the combined result of the dendrite availability and reliability problems is to force the lowering of dendritic firing and learning thresholds (*θ_F_* and *θ_Lpre_*), producing higher rates of background firing to untrained patterns (*P_F_*) as well as higher probabilities of dendritic learning (*P_L_*) and correspondingly higher dendrite usage (*µ_LD_*).

How does the lowering of learning thresholds affect synapse usage *µ_LS_*? Our intuition might be that increased dendrite usage leads to increased synapse usage but note that synapse usage is the product of three factors: dendrite usage *µ_LD_*, dendrite size *K*, and fraction of synapses potentiated per dendrite per learning event *f_pot_*. *µ_LD_* increases for short dendrites (Fig. 5e) and *K* decreases (by definition), but we must investigate *f_pot_*. *f_pot_* may be thought of as a measure of “dendrite inefficiency”, where higher values correspond to dendrites that must modify proportionally more synapses to encode a memory, decreasing the length of their age queue (*L*) and in turn decreasing the capacity of the network. *f_pot_* is approximately proportional to the ratio of the pre-synaptic learning threshold to the mean pre-synaptic activation, since $f_{pot}=\frac{\mu_{pot}}{K}\approx\frac{\theta_{Lpre}}{K\cdot\mu_{burst}}=f_{A}\frac{\theta_{Lpre}}{f_{A}\cdot K\cdot\mu_{burst}}=f_{A}\frac{\theta_{Lpre}}{\mu_{pre}}$ , where *µ_pre_* is the mean of *a_pre_*. This proportionality will allow us to understand how *f_pot_* varies with dendrite size by examining how the distribution of *a_pre_* varies with dendrite size. As seen in the progression from Fig. S4 a to b, the distributions of *a_pre_* are relatively broader for short dendrites than for long ones. For a given desired value of $P_{L}$ (we use 1% to illustrate the point), the greater variation in these response distributions for short dendrites forces a higher threshold relative to the mean ($\frac{\theta_{Lpre}}{\mu_{pre}}$) (see colored arrows indicating the 1% thresholds in Fig. S4b), producing higher values of *f_pot_* for short dendrites (Fig. S4c). If we conceptually multiply the *f_pot_* curve, the dendrite usage curve, and the implicit decreasing *K* curve (an exponential on the logarithmic plots used throughout this paper), we obtain the approximately constant synapse usage seen in Fig. 5e. In summary, as dendrites become smaller, the number of synapses used per dendrite does go down, which is helpful, but the increased dendrite usage, and decreased dendrite efficiency, conspire to produce approximately constant total synapse usage per pattern.

Naively, one would think that capacity would be tied to synapse use per pattern, but capacity continues to decrease despite constant synapse usage for small $K$. Why is this?

Here we turn to the analytical model developed earlier (Eq. 2). By rewriting Eq. 2 as a function of *f_pot_*, we will show that decreased dendrite efficiency explains the decrease in capacity for short dendrites. To do so, we use that $\mu_{LS}=\mu_{pot}\cdot P_{L}\cdot M=\mu_{pot}\cdot P_{L}\cdot N_{S}/K$, approximate *µ_pot_* by $\frac{\theta_{Lpre}}{\mu_{burst}}$ and *f_pot_* by $\frac{\theta_{Lpre}}{K\cdot\mu_{burst}}$, solve for *P_L_*, and insert our results into Eq. 2, yielding:

|  | $C=\left( \frac{f_{pot}\cdot N_{S}}{\mu_{LS}} \right)\left( \frac{\log(1-f_{S})}{\log\left( 1-f_{pot} \right)}-1 \right)$ |  |
| --- | --- | --- |

The term in parenthesis on the left is again the dendritic learning interval, 1/*P_L_* (i.e. the expected number of training patterns between learning events in a dendrite), written as a function of *f_pot_*, while the term on the right is the length of the age queue (*L*) as a function of *f_pot_*. For increasing values of *f_pot_*, the learning interval increases linearly, slowing the aging of potentiated synapses, and correspondingly extending memory lifetime. However, age queue length decreases more quickly than *P_L_* in the range of values of *f_pot_* in which we are interested, leading to a net decrease in memory capacity as *f_pot_* increases.

Using the observed constant value of *µ_LS_* from Fig. 5e (~150) and the observed dendrite efficiency curves from Fig. S4c, we can see that capacity indeed decreases almost linearly with increasing *f_pot_* (Fig. S4d). Re-plotting equation 3 as a function of *K* using the curve from Fig. S4c, we can compare our predicted capacity to that attained in the simulations (Fig. S4d). The qualitative match for small *K* indicates that shortened age queues are indeed a reason that capacity is reduced for short dendrites even if synapse usage remains constant.

In summary, we have seen why short dendrites are sub-optimal for memory capacity. As dendrites grow smaller, dendrite usage increases to overcome dendrite reliability problems, and as a side effect of the lowered learning and response thresholds needed to cope with the dendrite availability problem. The simultaneous decrease in dendrite size and efficiency roughly cancel to produce a constant usage of synapses. Despite this constant usage of synapses for short dendrites, the shortened age queues inside of those dendrites nevertheless result in decreased memory capacity.

Now that we understand the main constraints that decrease memory capacity for very short and very long dendrites, which explains the optimality of medium-sized dendrites, we can explain the relationship of the basic capacity curve to statistics of the input, including activation density and noise.

Increased Activation Density Decreases Capacity and Favors Short Dendrites

We saw in Figs. 4 and 5 that increased activation density decreases peak memory capacity, favors small dendrites, and leads to decreased dendrite usage for small and intermediate dendrite sizes. Intuitively, we might reason that, for a fixed dendrite size, increased activation density leads to increased synapse usage and thus should lower capacity. However, Fig. 4a reveals that, while capacity for networks with intermediate and long dendrites does decrease with increasing activation density, capacity for networks with short dendrites actually *increases* over some ranges of activation densities (e.g. from 1.5% to 3% in Fig. 5). A more sophisticated viewpoint based on the constraints identified in the previous two sections offers an explanation.

Recall that short dendrites suffer from unreliable readout – since memory features are encoded in fewer synapses than in long dendrites, dendritic post-synaptic activation *a_post_* displays larger fluctuations relative to its mean (Fig. S4b), leading to noisier signals within dendrites and an increased rate of readout failures. Increased activation density partially mitigates this effect by increasing the number of synapses activated in each dendrite, decreasing fluctuations relative to the mean in *a_post_*, and decreasing the rate of readout failures. Since each dendrite then provides a more reliable contribution to the memory trace, increased activation density allows networks with short dendrites to use fewer dendrites to store each memory (note the reduced dendrite usage in Fig. 5e). This decreased dendrite usage more than makes up for the increase in synapses usage per dendrite and explains the small gain in memory capacity for networks with short dendrites as activation density is increased.

Increased activation density is less kind to intermediate and long dendrites. Decreased dendrite usage causes the minimal encoding strength bound to become active earlier (that is, for shorter dendrites), leading to a shift of peak capacity towards shorter dendrites. For fixed dendrite sizes above the optimal value (i.e. those in which the minimal encoding strength bound is active), increased activation density simply results in increased synapse usage. At these dendrite sizes, readout failures are not a major constraint and thus do not offer the opportunity for the mitigating effects that occur in short dendrites.

Noise Decreases Memory Capacity and Favors Long Dendrites

We noted above that increased noise increases dendrite usage and decreases memory capacity in networks with short dendrites, and shifts optimal arbor morphology towards long dendrites. Intuitively, we expect noise to have a detrimental effect on memory performance, but why are these effects particularly concentrated in networks with short dendrites? Again, we may turn to the constraints identified in earlier sections.

Increased burst noise increases fluctuations relative to the mean in both *a_pre_* and *a_post_*. The increased variability in *a_pre_* increases the fraction of synapses *f_pot_* each dendrite must potentiate during a learning event, shortening age queues, accelerating aging, and decreasing memory capacity. The increased variability in *a_post_*, on the other hand, corresponds to noisier readout signals in dendrites, leading to increased rates of readout failure, increased dendrite usage (to overcome the unreliability of individual dendrites), and decreased memory capacity. Long dendrites are less susceptible to the detrimental effects of noise since, by summing over a larger number of synapses, fluctuations relative to the mean in *a_pre_* and *a_post_* grow more slowly with increasing noise. Note that while increased activation density had a mix of positive and negative effects, depending on dendrite size, increased noise has only negative consequences for this model of recognition memory.

Calculating an Upper Bound on per-Pattern Storage Cost in a Recognition Memory with Given Error Tolerances

Unlike a recall memory, a familiarity-based recognition memory does not store patterns *per se*, only memory traces strong enough to reliably distinguish familiar patterns from untrained background patterns. Here we calculate the per-pattern storage cost for a simple (non-neural) familiarity-based online recognition memory, thereby establishing an upper bound on the per-pattern storage cost for this type of memory. The analysis leads to the surprising conclusion that the per-pattern storage cost in this type of memory is both remarkably low, and independent of pattern size and density.

We assume that the input patterns are bit strings of length $N$, with $f_{A}N$ of the bits active. We construct a memory that is an ordered collection of $C$ “slots”, each of which contains a partial record of a single stored pattern, consisting of the first $n$ bits of the pattern. The network records memories by cycling through the slots, so that a newly “learned” pattern is always placed in the least-recently-used slot. When testing whether a pattern $p_{test}$ is familiar or not, the network responds “yes” if $p_{test}'s$ partial pattern matches any stored partial pattern, and “no” otherwise. Obviously, the network must store a large enough partial pattern for each learned pattern so that the probability of an accidental match between a random, untrained test pattern and *any* stored partial pattern is less than or equal to the allowable false positive error rate $\epsilon_{+}$. When this constraint is met, the network functions as a $C$-capacity recognition memory with false positive rate ${= \epsilon}_{+}$ and false negative rate of $\epsilon_{-}= 0$. (A simple adjustment to the calculation can be made to accomodate a non-zero false negative rate $\epsilon_{-}$, but this will have no effect on our general conclusions, so for simplicity we stick to the most conservative assumption of a zero false negative rate).

To calculate the false positive rate for given $N, f_{A}, C, n, \epsilon_{+}$, we first calculate the probability that a random, untrained pattern will match a given stored partial pattern. Since the stored partial pattern could be any one of the $\left( \begin{matrix} n \\ f_{A}n \end{matrix} \right)$ strings of $n$ bits with $\approx f_{A}n$ active bits, the probability of matching one untrained pattern will be $\approx1/\left( \begin{matrix} n \\ f_{A}n \end{matrix} \right)$. We can then write an expression for the probability that an untrained pattern matches any one of the $C$ stored partial patterns, and set it equal to the false positive rate $\epsilon_{+}$.

$$\epsilon_{+}= 1-\left( 1- \frac{1}{\left( \begin{matrix} n \\ f_{A}n \end{matrix} \right)} \right)^{C}$$

To estimate the number of bits of information required to store each partial pattern, one might naively solve for $n$, the bit-length of the partial patterns. However, the true information content of each partial pattern is $\log_{2} \left( \begin{matrix} n \\ f_{A}n \end{matrix} \right)$. Solving the equation above for $\left( \begin{matrix} n \\ f_{A}n \end{matrix} \right)$ and taking logs, we have the per-pattern cost in bits:

$$B{= log}_{2} \left( \begin{matrix} n \\ f_{A}n \end{matrix} \right)=-\log_{2} \left( {1- \left( 1-\epsilon_{+} \right)}^{1/C} \right)$$

For small $\epsilon_{+}$, this simplifies to $B{= log}_{2} \left( \frac{C}{\epsilon_{+}} \right)$.

Surprisingly, the per-pattern storage cost is independent of the size or density of the input patterns. That is, no matter how large or how sparse/dense the input patterns are, it takes a constant number of bits per pattern to store $C$ patterns in an online memory subject to a given error tolerance.

As an example, assuming a capacity of $C= 30,000$ memories and a false positive rate of $\epsilon_{+}=1\%$, we have that the per-pattern storage cost is $B\approx21.5$ bits.

Variability Reduces Efficiency in a Recognition Memory

Analogous to the above calculation that shows that only a small fragment of a binary pattern needs to be stored in a recognition memory to achieve a low recognition error rate, we may ask how few dendrites must encode a pattern in a dendrite-based memory, and whether this number, too, is both very small and independent of pattern size or density. In the limit, one might imagine that a recognition memory could function by learning in just one dendrite if (1) that dendrite is capable of driving its parent neuron to spike and (2) the network is capable of reliably suppressing any response to untrained patterns. This line of thought leads us to the rather surprising conclusion that a dendrite-based recognition memory might be able to work in an extraordinarily sparse regime.

Let us imagine a network which does indeed aim to encode each pattern in a single dendrite. We will also temporarily assume that dendrites that encode a pattern respond with perfect reliability upon an immediate, second presentation of that pattern, so that there are no immediate “readout failures”, and the mean, immediate response is equal to the mean number of dendrites that learned that pattern (*µ_LD_* for “learning dendrites”). Under this assumption, the spread in the distribution of responses to learned patterns is due entirely to variability in the number of dendrites that learn a pattern (as opposed to variability in the *responses* of learned dendrites), and the exact form of the distribution is a binomial distribution *β(M, P_L_)*, where *P_L_* is the probability that an arbitrary dendrite learns in response to a given pattern, $M$ is the total number of dendrites in the system, and the mean number of dendrites used is $\mu_{LD}=P_{L}*M$. Furthermore, since *P_L_* is small and $M$ large, this distribution is well-approximated by a Poisson distribution with mean (and variance) *µ_LD_*. As a Poisson distribution is fully characterized by its mean, the distribution of learned responses is determined only by *µ_LD_*, and for a fixed mean, we have no parameters left to change the variance. Now we can see why a small *µ_LD_* is not possible: too many patterns will fail to trigger learning in even a single dendrite (hereafter “learning failure” or “encoding failure”). For example, if *µ_LD_* = 1 (Fig. 6a top), 37% of patterns will not be encoded by a single dendrite, those so-called “trained” patterns will later be indistinguishable from untrained patterns (since nothing was learned), and the false negative error rate will be *at least* 37%. Since we chose an error tolerance of $\epsilon_{+}=1\%$*,* this outcome is unacceptable. In order to avoid rampant encoding failures and drive the immediate miss rate below 1%, we must set $\mu_{LD}\geq5$. Thus, the probabilistic nature of memory, and the resulting variability in the strength of the stored trace, sets a lower bound on the trace strength a recognition memory can possibly aim to use (unless an explicit response normalization system is employed). Still, this lower bound of just a handful of dendrites suggests the possibility of an astonishingly sparse operating regime on the scale of a brain area.

However, there are two additional complications that must be considered. First, input noise can cause immediate readout failures in some fraction of dendrites whose active axons happen to receive unusually small spike bursts. Second, given that synapses encoding any particular pattern are distributed across different dendrites, random variations in the learning rates of different dendrites will lead to variations in the aging rates of the synapses that make up the memory, so that the memory trace for a single pattern will lose dendrites gradually rather than abruptly as it nears the end of its storage lifetime. Given these two effects, the optimization of learning/firing thresholds typically leads to an encoding strength above the in-principle minimum encoding bound of 5 (Figure 6a), to $\mu_{LD}\approx7$, as seen in the simulation runs of Fig. 5c,d in the mid-to-long dendrite range.

Novelty vs. Familiarity Neurons

In the perirhinal cortex, the area most closely associated with familiarity-based recognition memory (as opposed to recall memory which is centered in the hippocampal formation; for reviews, see [2–4]), it has been reported that the majority of neurons that show familiarity-related responses respond *less* on a repeat presentations of a pattern compared to its first presentation, while response increases to previously seen patterns are very rare (i.e. seen in less than 1% of neurons) [5,6]. If capacity maximization has been a driving force in the evolution of neural memory systems, then the finding that most familiarity-related neurons signal "inverse familiarity" is conceptually surprising for the following reasons. If it were literally true that the great majority of familiarity-related neurons in the PRC respond at high firing rates to most or all novel stimuli, thereby setting a high baseline, and then they lower their responses to familiar patterns, then we face a quandary. As we have seen, in order to maximize storage capacity in an online learning system, only very few synapses should encode each pattern, and if responses are to go *down* with learning, then it follows that those few synapses that change during learning in such an inverse system should undergo weakening rather than strengthening. If only a few synapses on a few (e.g. 10) neurons or dendrites are weakened during the learning of a pattern (the inverse of the situation that we model), then in a memory network containing 10,000 neurons that all fire to all novel patterns, the response to a familiar pattern would drop to 9,990 neurons, which is virtually indistinguishable from the high baseline. In short, the system would suffer from an untenable signal to noise problem. If as an alternative, a much large number of the (inverse) familiarity neurons were “taught” to lower their firing rates to every familiar stimulus, this implies that synaptic changes would have to occur on a much larger number of neurons each time a pattern is learned, which conflicts with the goal to minimize the use of storage resources.

One way to reconcile the observation that most recorded neurons in a neurophysiological experiment show response reductions to familiar patterns, whereas neurons showing response increases are very rare, is to posit that, (1) as previously discussed, the small number of neurons (e.g. 10) involved in encoding each pattern are difficult to detect using traditional neurophysiological methods – especially considering that these neurons would be classified as visually unresponsive when probed with novel test patterns; and (2) neurons that systematically respond to novel patterns might be inhibitory interneurons that do not encode memories *per se*, but are part of a local response normalization system. According to this view, a sizable population of inhibitory normalization neurons, perhaps accounting for 10% of all neurons in the area, would be excited by *every* input pattern via conventional activity pooling mechanisms [7], but then be transiently inhibited by disinhibitory interneurons (e.g. VIP interneurons [8,9]) whenever a sufficient number (e.g. 10) of the principal neurons in the area is strongly activated by a familiar pattern. Imaging studies with genetically encoded indicators that allow different classes of neurons to be visualized while responding to novel and familiar patterns would be the ideal way to sort out this issue.

## SUPPORTING INFORMATION REFERENCES

1. Brady TF, Konkle T, Alvarez GA, Oliva A. Visual long-term memory has a massive storage capacity for object details. PNAS. 2008;105: 14325–14329. doi:10.1073/pnas.0803390105

2. Bogacz R, Brown MW. Comparison of computational models of familiarity discrimination in the perirhinal cortex. Hippocampus. 2003;13: 494–524. doi:10.1002/hipo.10093

3. Brown MW, Aggleton JP. Recognition memory: what are the roles of the perirhinal cortex and hippocampus? Nature Reviews Neuroscience. 2001;2: 51–61.

4. Eichenbaum H, Sauvage M, Fortin N, Komorowski R, Lipton P. Towards a functional organization of episodic memory in the medial temporal lobe. Neuroscience & Biobehavioral Reviews. 2012;36: 1597–1608. doi:10.1016/j.neubiorev.2011.07.006

5. Sohal VS, Hasselmo ME. A model for experience-dependent changes in the responses of inferotemporal neurons. Network: Computation in Neural Systems. 2000;11: 169–190.

6. Xiang JZ, Brown MW. Differential neuronal encoding of novelty, familiarity and recency in regions of the anterior temporal lobe. Neuropharmacology. 1998;37: 657–676.

7. Carandini M, Heeger DJ. Normalization as a canonical neural computation. Nature Reviews Neuroscience. 2012;13: 51–62. doi:10.1038/nrn3136

8. Karnani MM, Jackson J, Ayzenshtat I, Hamzehei Sichani A, Manoocheri K, Kim S, et al. Opening Holes in the Blanket of Inhibition: Localized Lateral Disinhibition by VIP Interneurons. J Neurosci. 2016;36: 3471–3480. doi:10.1523/JNEUROSCI.3646-15.2016

9. Pi H-J, Hangya B, Kvitsiani D, Sanders JI, Huang ZJ, Kepecs A. Cortical interneurons that specialize in disinhibitory control. Nature. 2013;503: 521–524. doi:10.1038/nature12676
