# Supplementary figures and images for: How Dendrites Affect Online Recognition Memory
Source: PLoS Comput Biol. 2019 May 3;15(5):e1006892. doi: 10.1371/journal.pcbi.1006892 (PMC6527246; doi:10.1371/journal.pcbi.1006892)

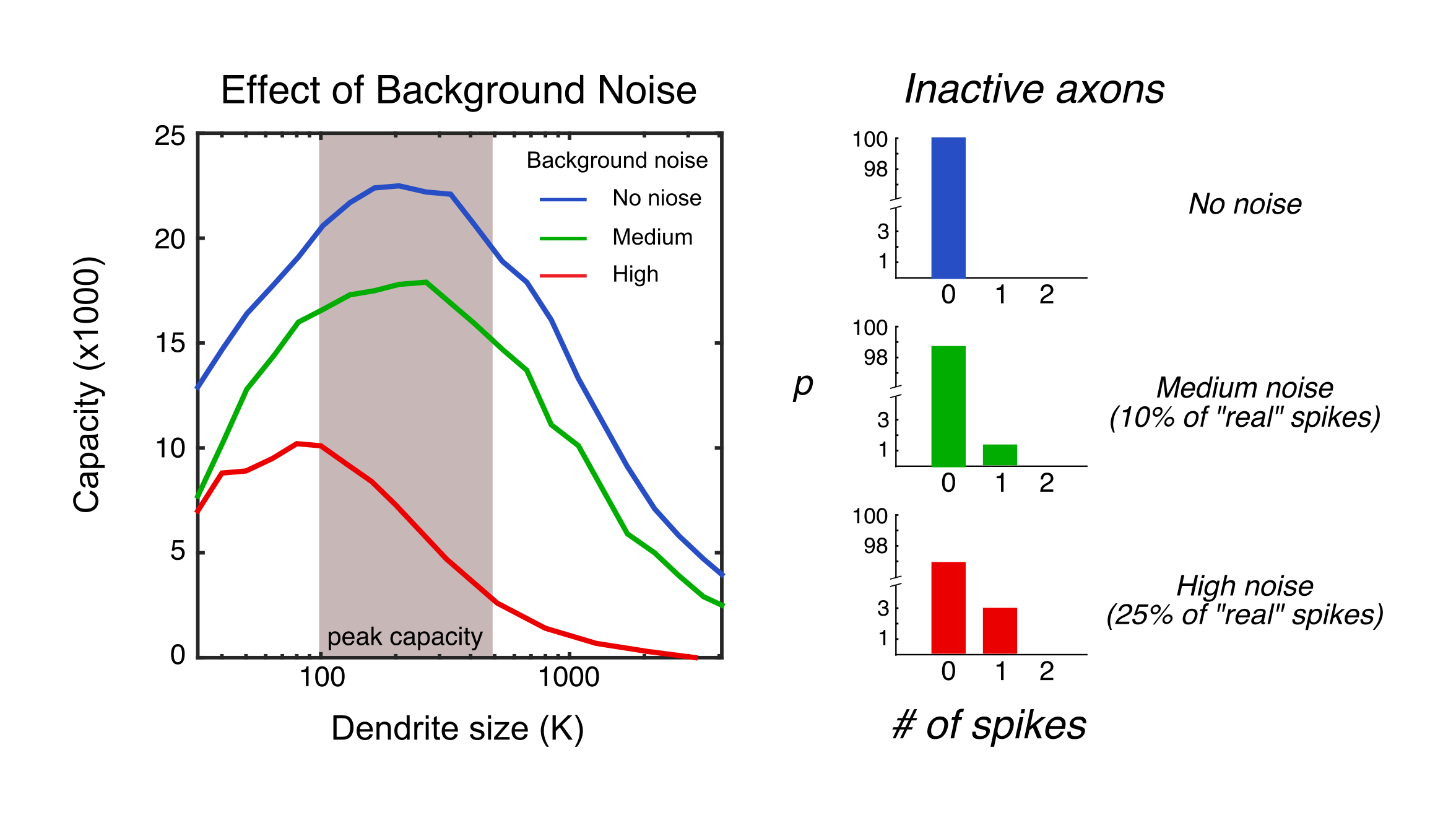

Supplement: S1 Fig — In the base case without background noise, nominally inactive axons (which were the vast majority) never fired. For the medium and high noise cases, nominally inactive axons emitted one spike with the indicated probability. The fraction of inactive axons that fired a spike was chosen so that in the medium case, aberrant spikes totaled approximately 10% of the number of “real” pattern spikes (recall that each active axon generated a burst of 4 spikes on average), and in the high noise case, aberrant spikes were 25% of the real spikes. Increasing background noise decreased memory capacity, and, at high noise levels, pushed the optimal dendrite size to shorter values. For all simulations here, the dendritic activation slope parameter was set to 3. (TIF) [file pcbi.1006892.s003.tif]

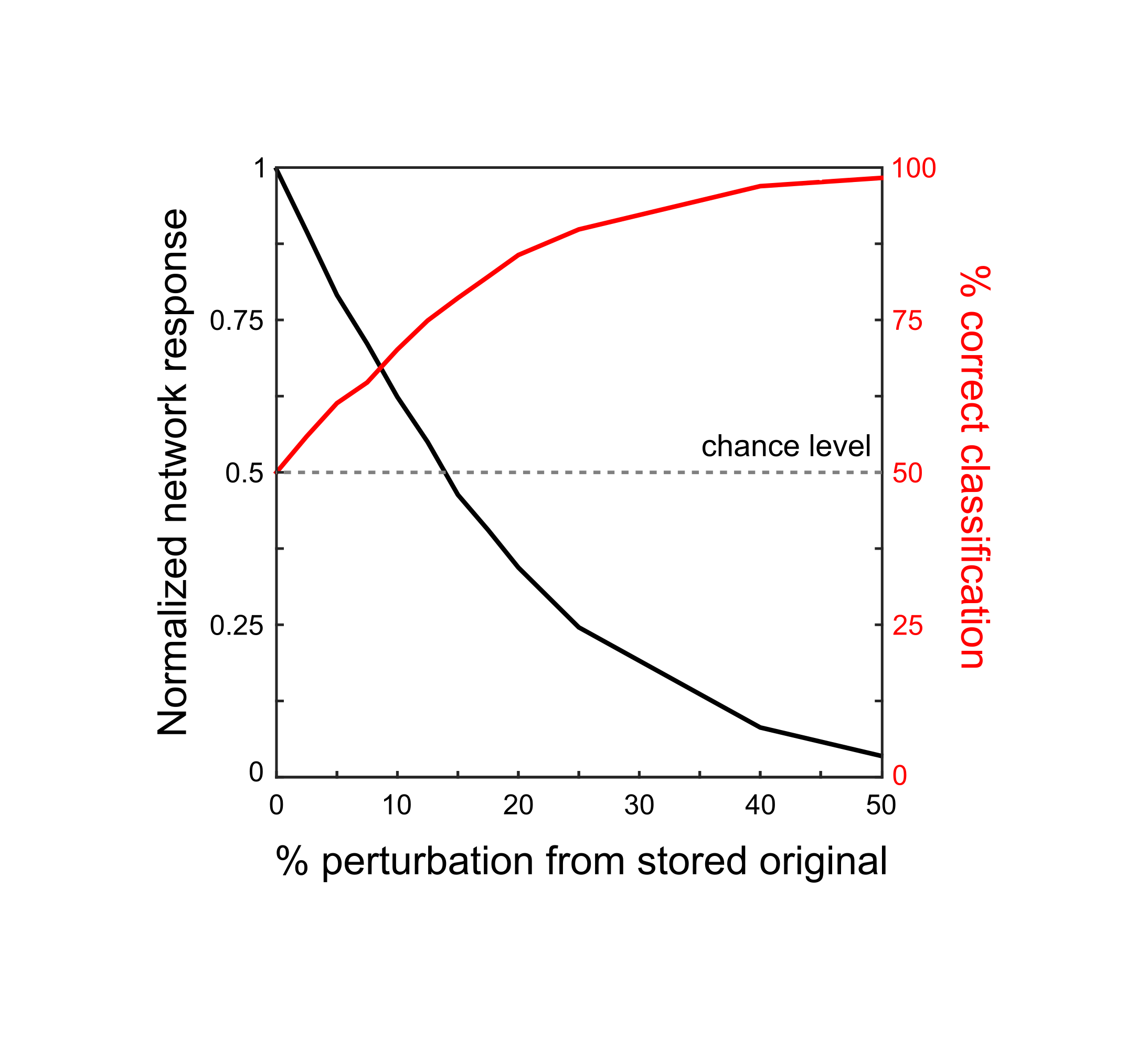

Supplement: S2 Fig — The memory network was trained as normal to maximize old/new recognition capacity. We then tested how a trained network responded to perturbed versions of stored patterns. As expected, as an increasing fraction of training pattern bits were changed, network response decreased (black curve). For example, when 20% of an original training pattern’s active bits were assigned to different input lines (keeping pattern density unchanged), average network response fell to roughly one third of the original response. We then tested whether the network could reliably distinguish between exact trained patterns and perturbed patterns (red curve). The network was able to distinguish exact training patterns from 20% perturbed patterns with 85% accuracy. (TIF) [file pcbi.1006892.s004.tif]

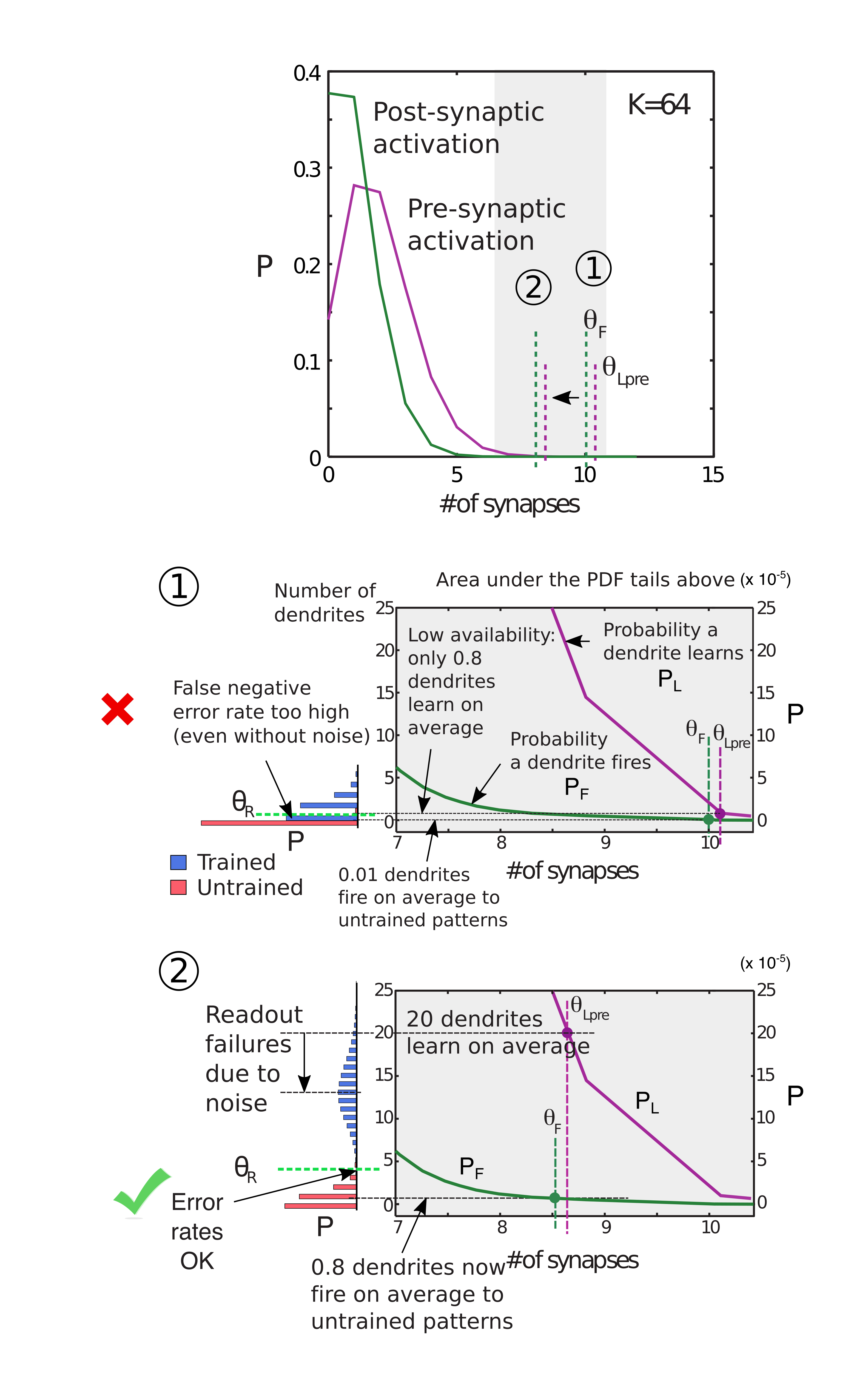

Supplement: S3 Fig — (See S1 Text for details). (TIF) [file pcbi.1006892.s005.tif]

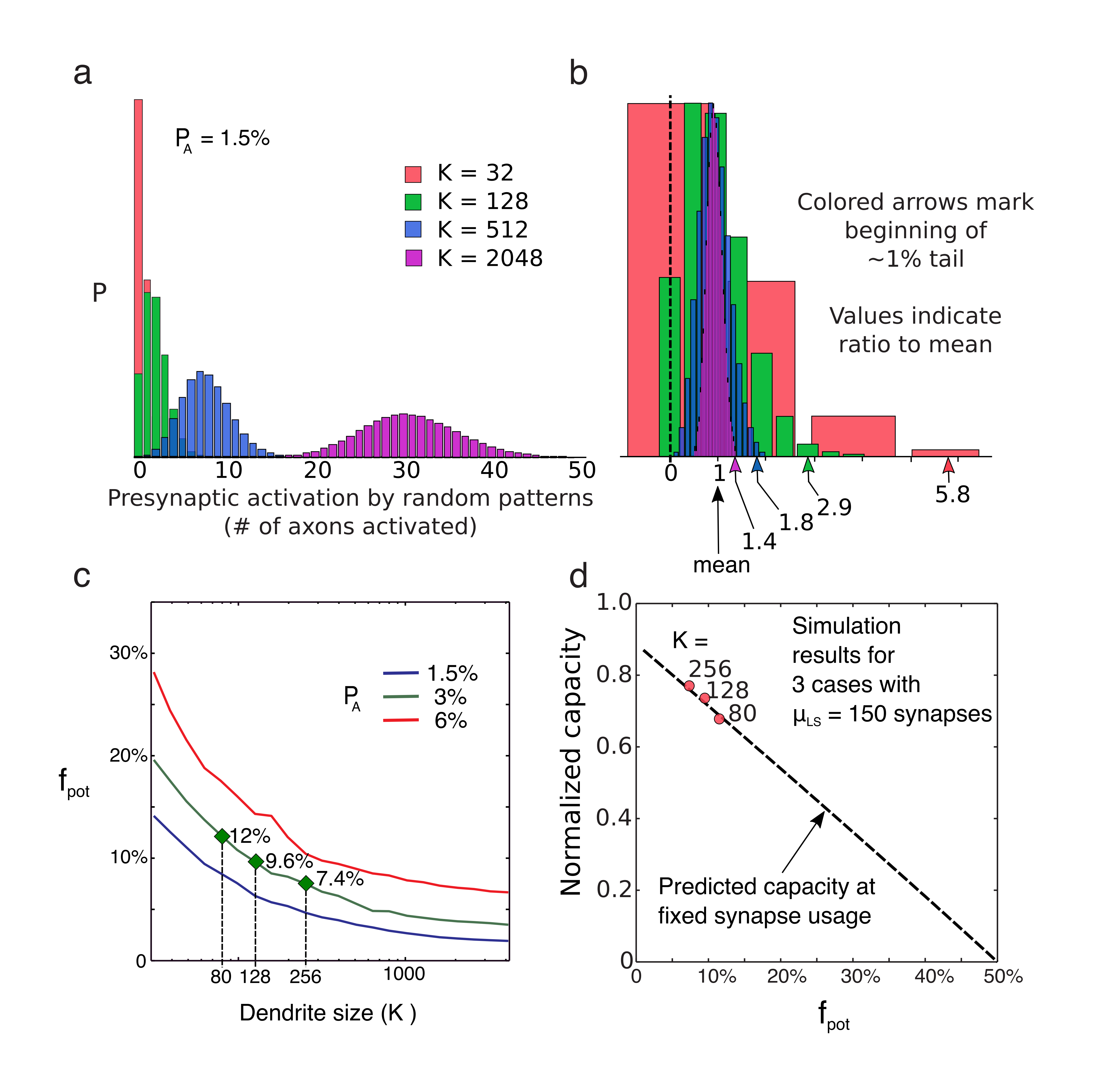

Supplement: S4 Fig — (a) Distributions of pre-synaptic responses to random patterns for dendrites of varying size. (b) Same graph as (a) but with responses normalized to the mean response. Colored arrows indicate points where the upper 1% of the probability mass begins, to illustrate that shorter dendrites have larger response variability relative to their mean than longer dendrites. (c) Fraction of synapses used within each dendrite involved in learning increases for short dendrites. (d) Comparison of capacity for 3 cases with equivalent synapse usage (red dots); capacity drops linearly for shorter dendrites because of the higher values of fpot. (TIF) [file pcbi.1006892.s006.tif]
