# Supplementary material for: How Dendrites Affect Online Recognition Memory
Source: PLoS Comput Biol. 2019 May 3;15(5):e1006892. doi: 10.1371/journal.pcbi.1006892 (PMC6527246; doi:10.1371/journal.pcbi.1006892)
Supplement: S1 Data — (ZIP) [file pcbi.1006892.s002.zip › Plos data:code/Figure data.zip!/Figure data/old/9.pdf]

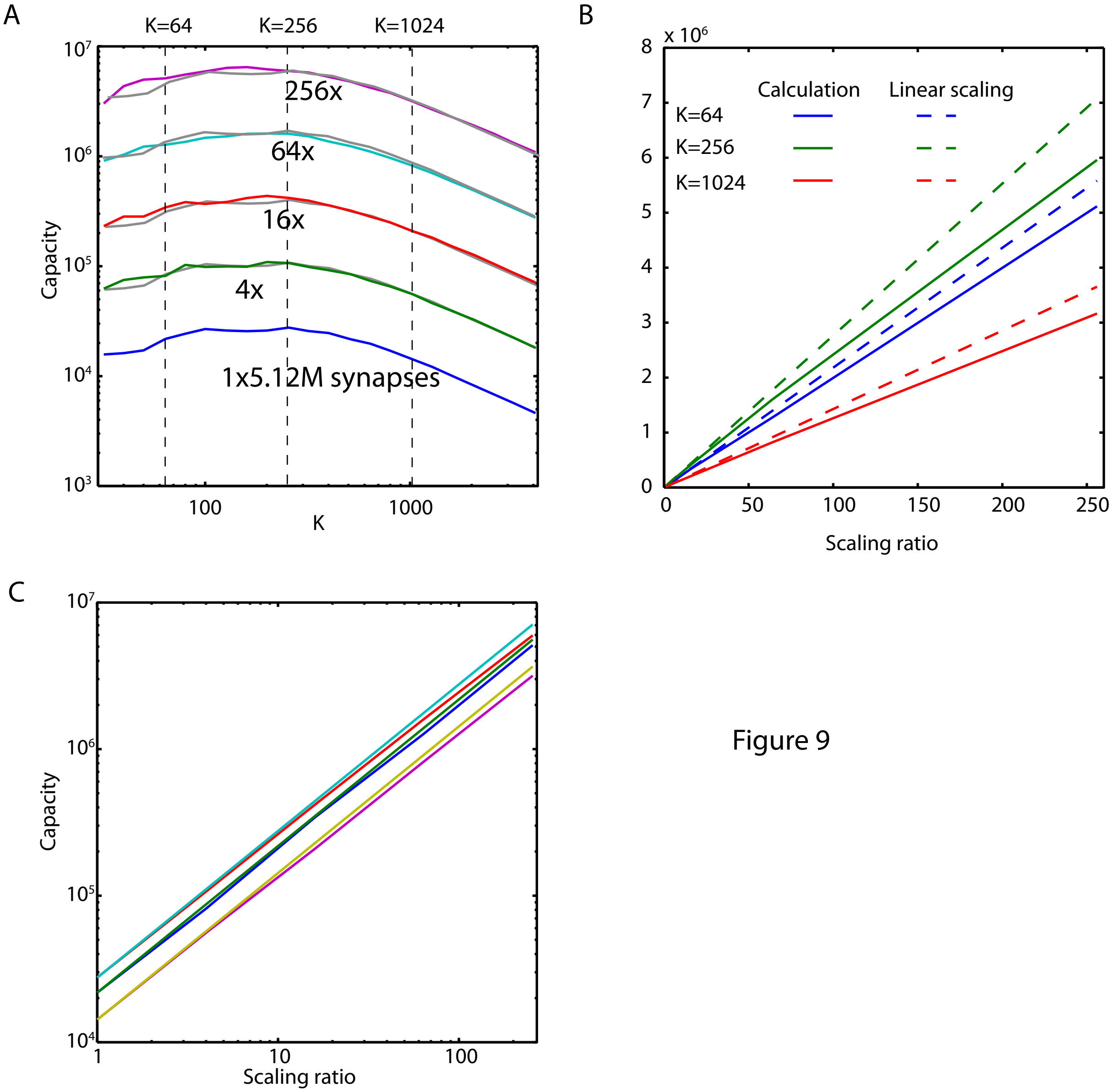
A
K=64
K=256
K=1024
Capacity
K
256x
64x
16x
4x
1x5.12M synapses
B
x 10^6
Calculation
Linear scaling
K=64
K=256
K=1024
Scaling ratio
C
Capacity
Scaling ratio

Figure 9
