# Supplementary material for: How Dendrites Affect Online Recognition Memory
Source: PLoS Comput Biol. 2019 May 3;15(5):e1006892. doi: 10.1371/journal.pcbi.1006892 (PMC6527246; doi:10.1371/journal.pcbi.1006892)
Supplement: S1 Data — (ZIP) [file pcbi.1006892.s002.zip › Plos data:code/Figure data.zip!/Figure data/old/8.pdf]

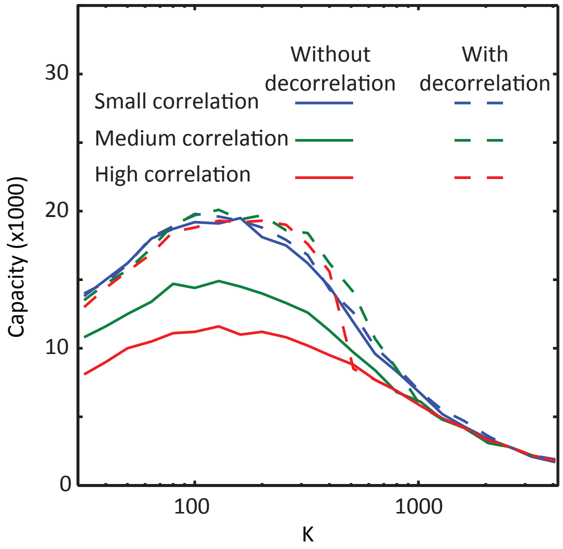

Without decorrelation
With decorrelation
Small correlation
Medium correlation
High correlation
Capacity (x1000)
0
10
20
30
100
1000
K

Figure 8.
