# Supplementary material for: How Dendrites Affect Online Recognition Memory
Source: PLoS Comput Biol. 2019 May 3;15(5):e1006892. doi: 10.1371/journal.pcbi.1006892 (PMC6527246; doi:10.1371/journal.pcbi.1006892)
Supplement: S1 Data — (ZIP) [file pcbi.1006892.s002.zip › Plos data:code/Figure data.zip!/Figure data/old/1-new.pdf]

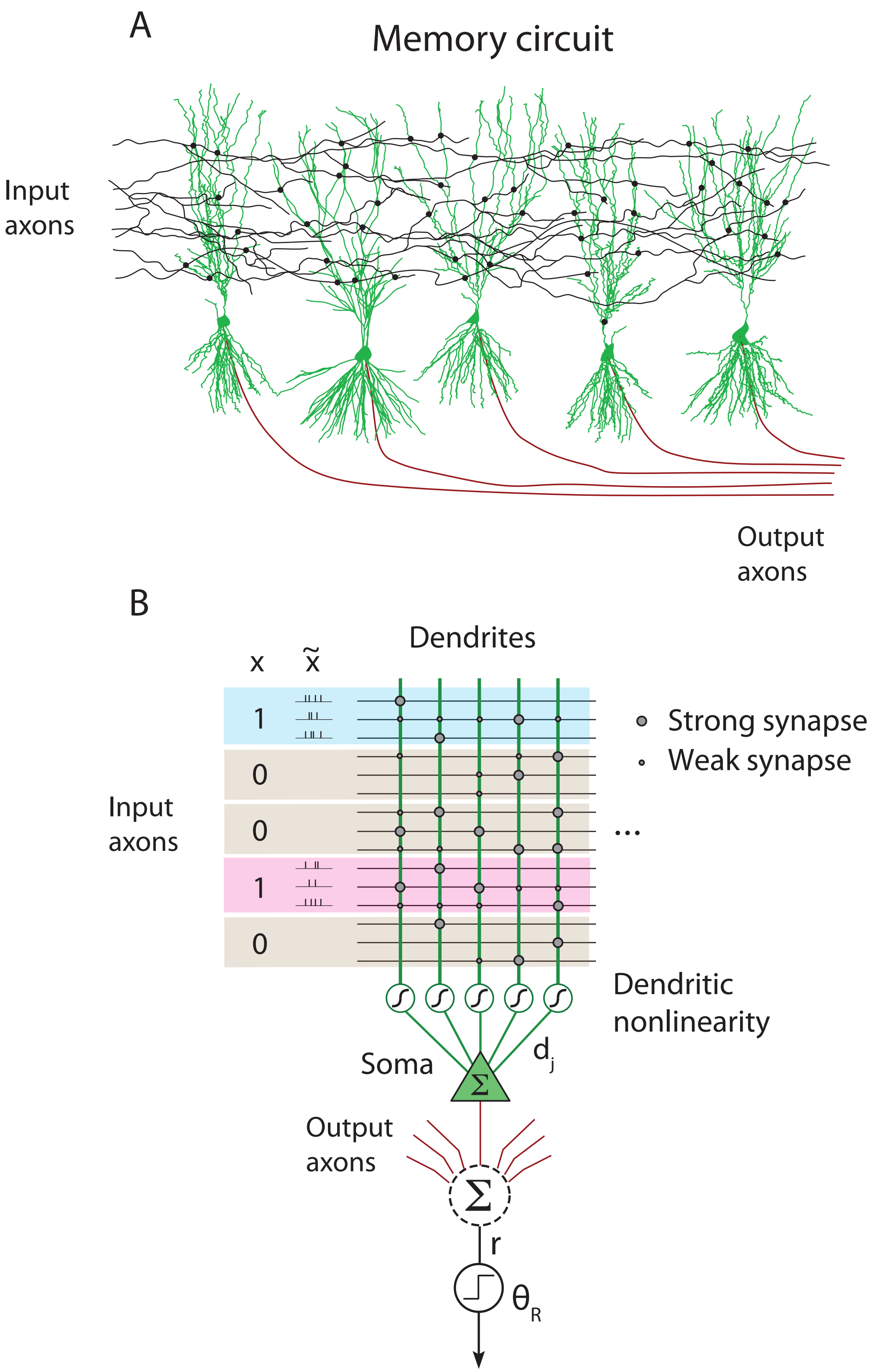
A
Memory circuit
Input
axons
Output
axons
B
Dendrites
x
x̃
1
0
0
1
0
Strong synapse
Weak synapse
Input
axons
...
Dendritic
nonlinearity
Soma
d_j
Σ
Output
axons
Σ
r
θ_R
