# Supplementary material for: How Dendrites Affect Online Recognition Memory
Source: PLoS Comput Biol. 2019 May 3;15(5):e1006892. doi: 10.1371/journal.pcbi.1006892 (PMC6527246; doi:10.1371/journal.pcbi.1006892)
Supplement: S1 Data — (ZIP) [file pcbi.1006892.s002.zip › Plos data:code/Figure data.zip!/Figure data/old/6.pdf]

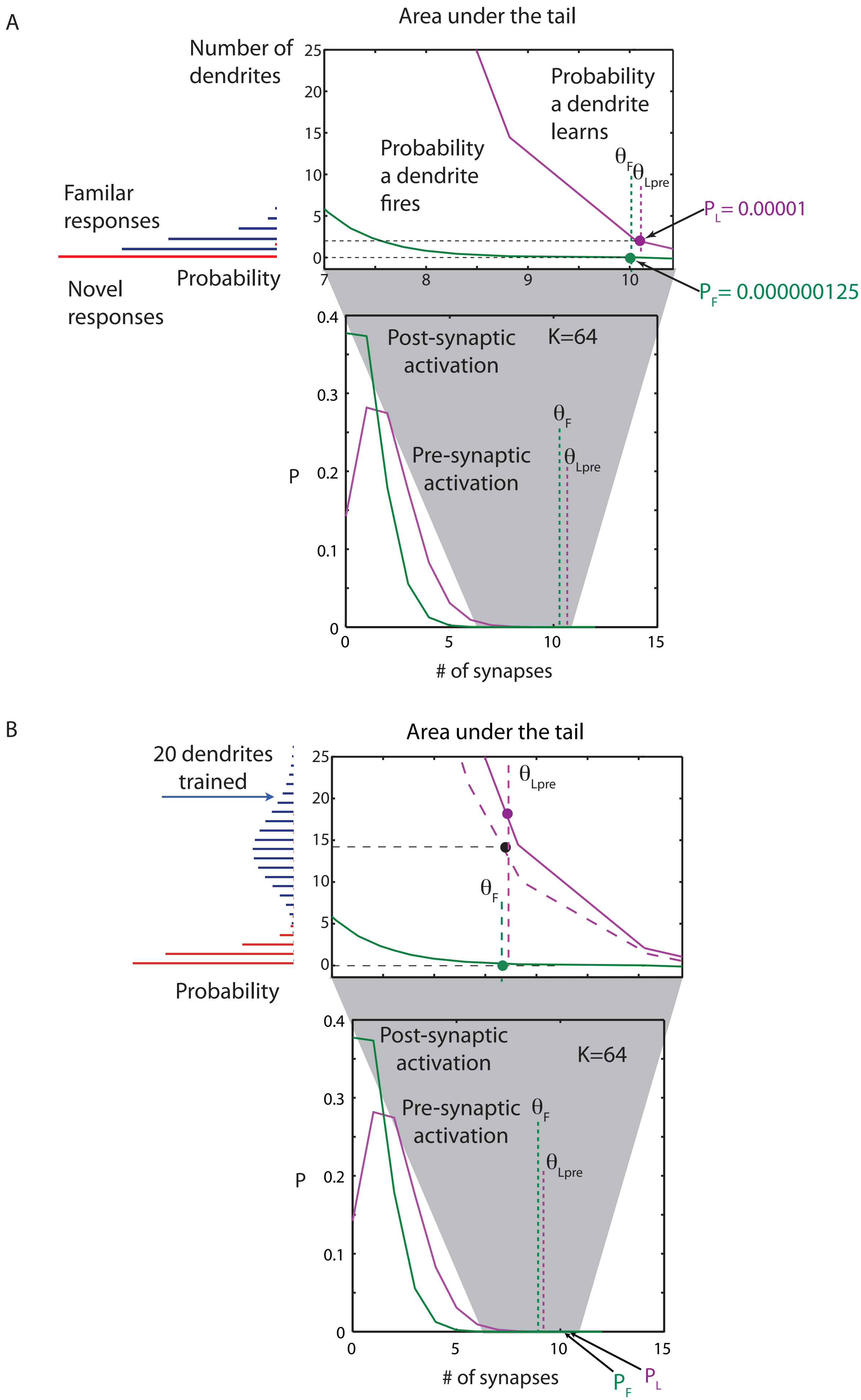
A
Area under the tail
Number of dendrites
Probability a dendrite learns
Probability a dendrite fires
Familar responses
Novel responses
Probability
P_L= 0.00001
P_F= 0.000000125
Post-synaptic activation
K=64
Pre-synaptic activation
P
# of synapses
B
Area under the tail
20 dendrites trained
Probability
Post-synaptic activation
K=64
Pre-synaptic activation
P
# of synapses

Figure 6
