# Supplementary material for: How Dendrites Affect Online Recognition Memory
Source: PLoS Comput Biol. 2019 May 3;15(5):e1006892. doi: 10.1371/journal.pcbi.1006892 (PMC6527246; doi:10.1371/journal.pcbi.1006892)
Supplement: S1 Data — (ZIP) [file pcbi.1006892.s002.zip › Plos data:code/Figure data.zip!/Figure data/old/7.pdf]

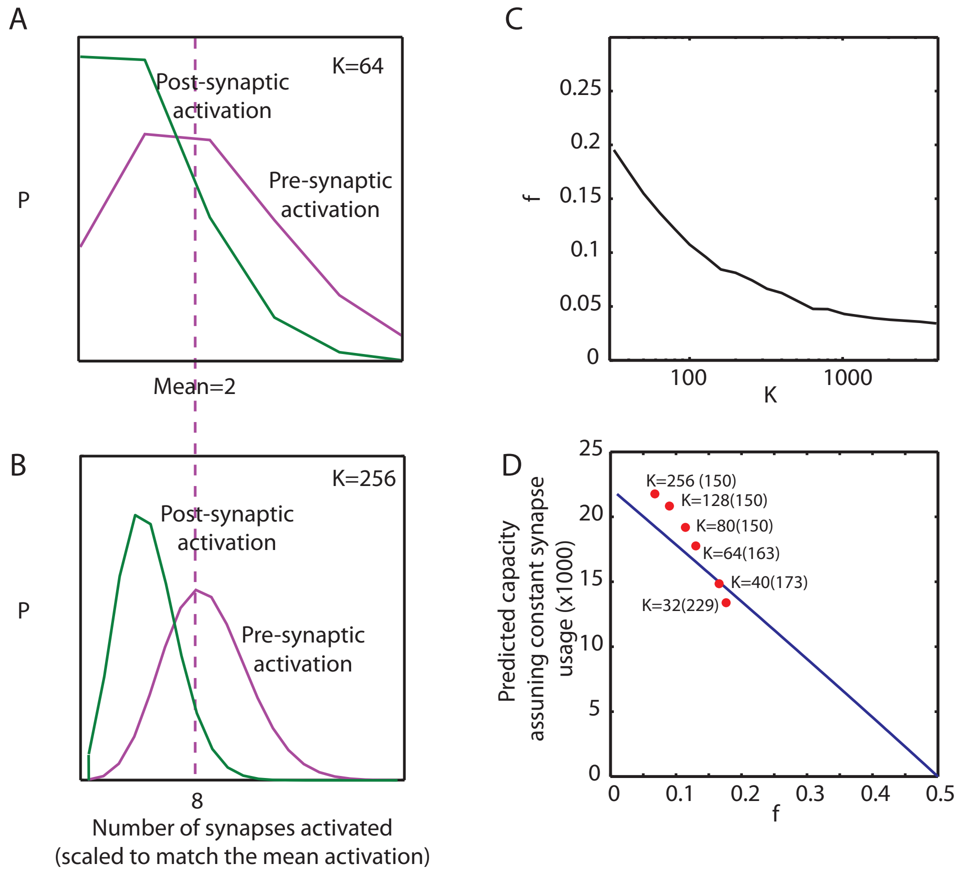

A
K=64
Post-synaptic activation
Pre-synaptic activation
P
Mean=2
B
K=256
Post-synaptic activation
Pre-synaptic activation
P
8
Number of synapses activated
(scaled to match the mean activation)
C
f
0.25
0.2
0.15
0.1
0.05
0
100
1000
K
D
Predicted capacity assuning constant synapse usage (x1000)
25
20
15
10
5
0
0
0.1
0.2
0.3
0.4
0.5
f
K=256 (150)
K=128(150)
K=80(150)
K=64(163)
K=40(173)
K=32(229)

Figure 7
