# Supplementary material for: How Dendrites Affect Online Recognition Memory
Source: PLoS Comput Biol. 2019 May 3;15(5):e1006892. doi: 10.1371/journal.pcbi.1006892 (PMC6527246; doi:10.1371/journal.pcbi.1006892)
Supplement: S1 Data — (ZIP) [file pcbi.1006892.s002.zip › Plos data:code/Figure data.zip!/Figure data/old/5.pdf]

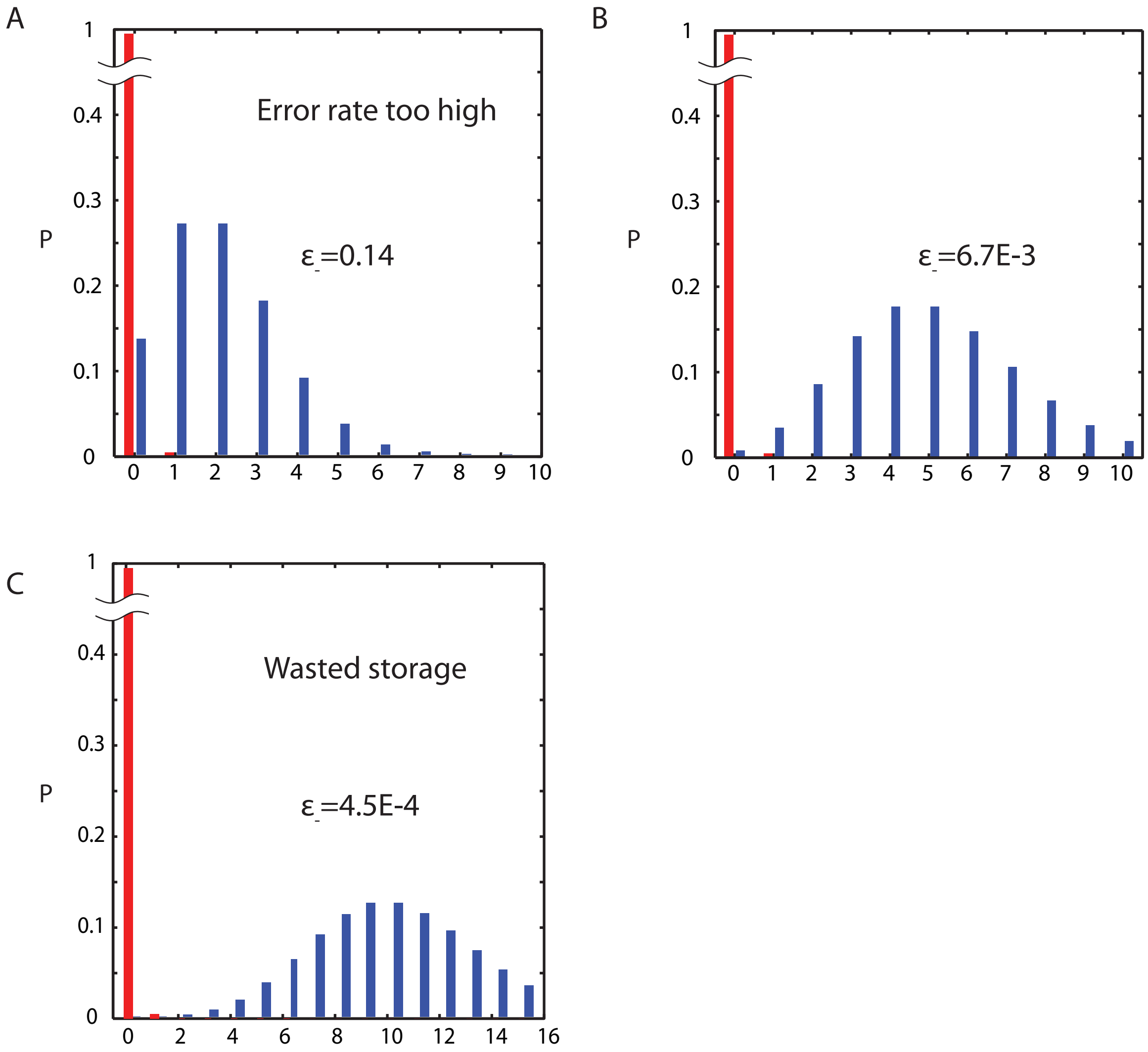
A
Error rate too high
ε-=0.14
B
ε-=6.7E-3
C
Wasted storage
ε-=4.5E-4
P
0
0.1
0.2
0.3
0.4
1
0 1 2 3 4 5 6 7 8 9 10
0 2 4 6 8 10 12 14 16

Figure 5.
