# Supplementary material for: How Dendrites Affect Online Recognition Memory
Source: PLoS Comput Biol. 2019 May 3;15(5):e1006892. doi: 10.1371/journal.pcbi.1006892 (PMC6527246; doi:10.1371/journal.pcbi.1006892)
Supplement: S1 Data — (ZIP) [file pcbi.1006892.s002.zip › Plos data:code/Figure data.zip!/Figure data/old/4.pdf]

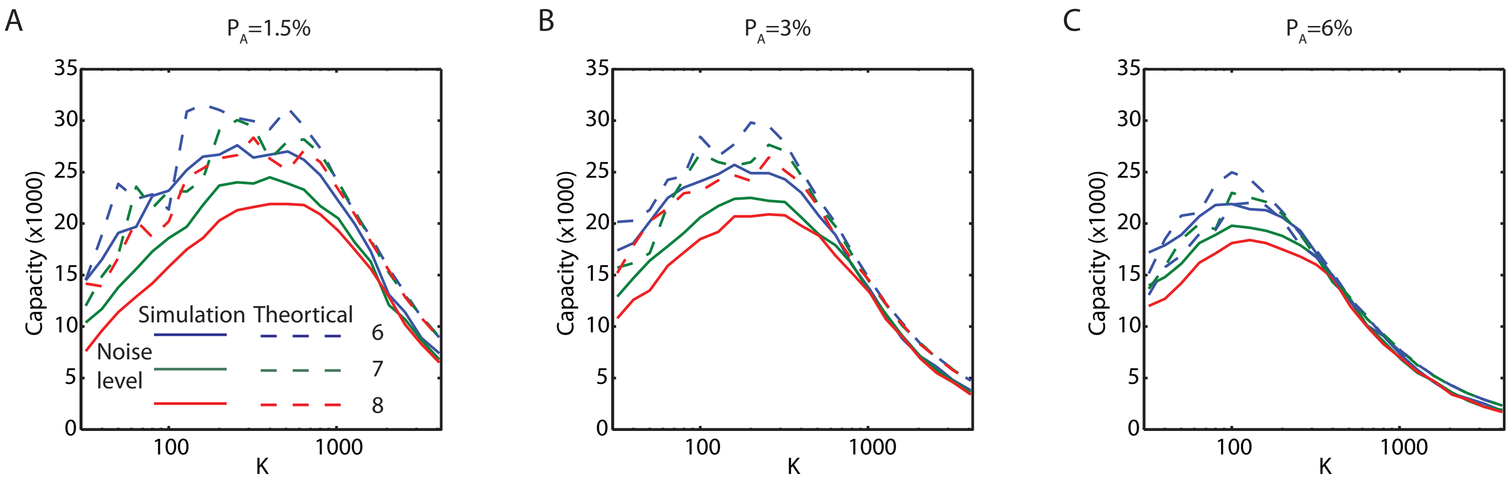

A
P_A=1.5%
B
P_A=3%
C
P_A=6%
Capacity (x1000)
K
Simulation
Theortical
Noise level
6
7
8
35
30
25
20
15
10
5
0
100
1000

Figure 4.
