# Supplementary material for: How Dendrites Affect Online Recognition Memory
Source: PLoS Comput Biol. 2019 May 3;15(5):e1006892. doi: 10.1371/journal.pcbi.1006892 (PMC6527246; doi:10.1371/journal.pcbi.1006892)
Supplement: S1 Data — (ZIP) [file pcbi.1006892.s002.zip › Plos data:code/Figure data.zip!/Figure data/old/1.pdf]

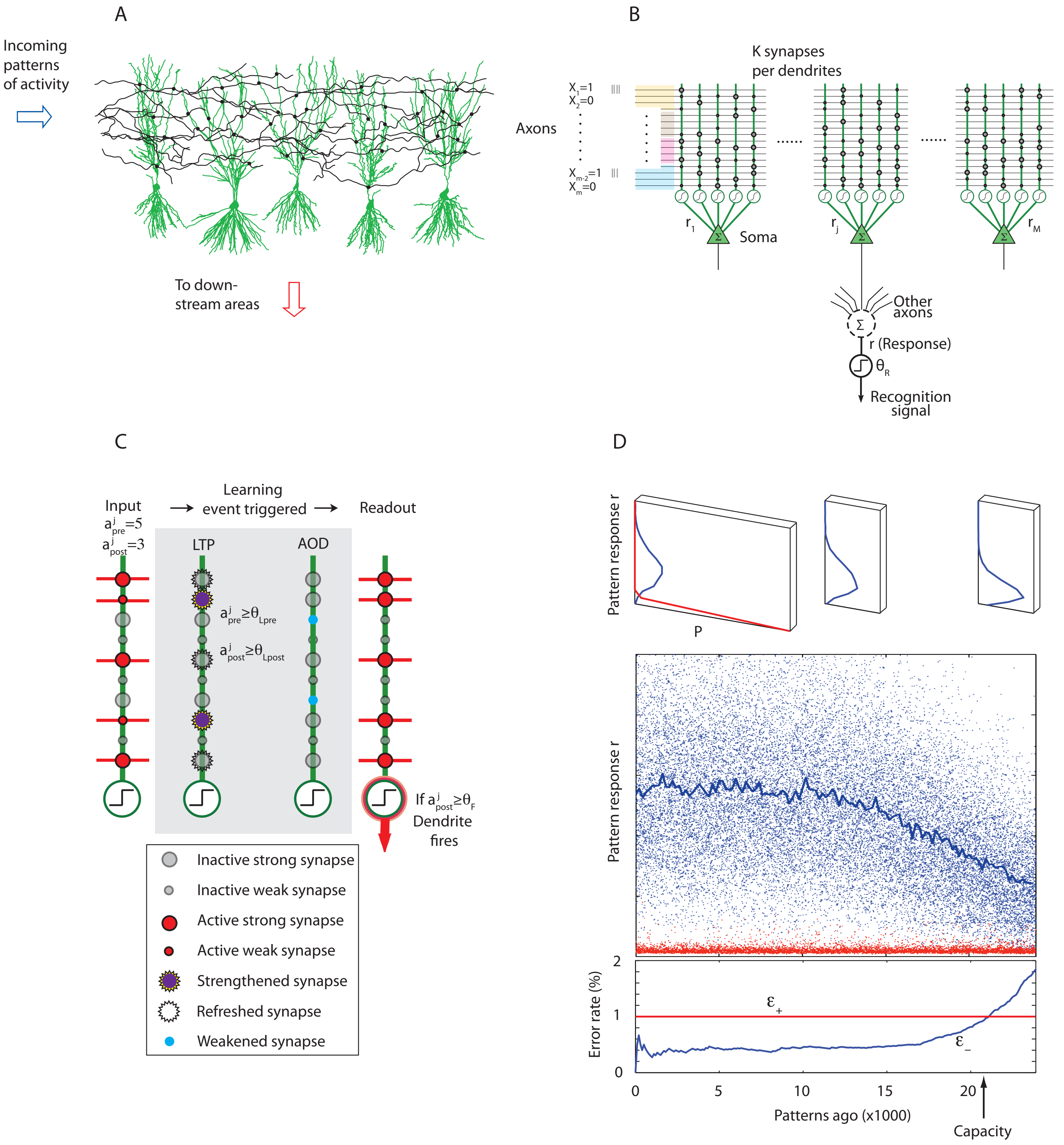
A
Incoming patterns of activity
To down-stream areas
B
K synapses per dendrites
Axons
X_1=1
X_2=0
X_{m-2}=1
X_m=0
r_1
r_j
r_M
Soma
Other axons
r (Response)
θ_R
Recognition signal
C
Input
a^j_{pre}=5
a^j_{post}=3
Learning event triggered
Readout
LTP
AOD
$a^j_{pre} \geq \theta_{Lpre}$
$a^j_{post} \geq \theta_{Lpost}$
If $a^j_{post} \geq \theta_F$ Dendrite fires
Inactive strong synapse
Inactive weak synapse
Active strong synapse
Active weak synapse
Strengthened synapse
Refreshed synapse
Weakened synapse
D
Pattern response r
P
Pattern response r
Error rate (%)
ε_+
ε_-
0
1
2
0
5
10
15
20
Patterns ago (x1000)
Capacity

Figure 1.
