# Supplementary material for: How Dendrites Affect Online Recognition Memory
Source: PLoS Comput Biol. 2019 May 3;15(5):e1006892. doi: 10.1371/journal.pcbi.1006892 (PMC6527246; doi:10.1371/journal.pcbi.1006892)
Supplement: S1 Data — (ZIP) [file pcbi.1006892.s002.zip › Plos data:code/Figure data.zip!/Figure data/old/3.pdf]

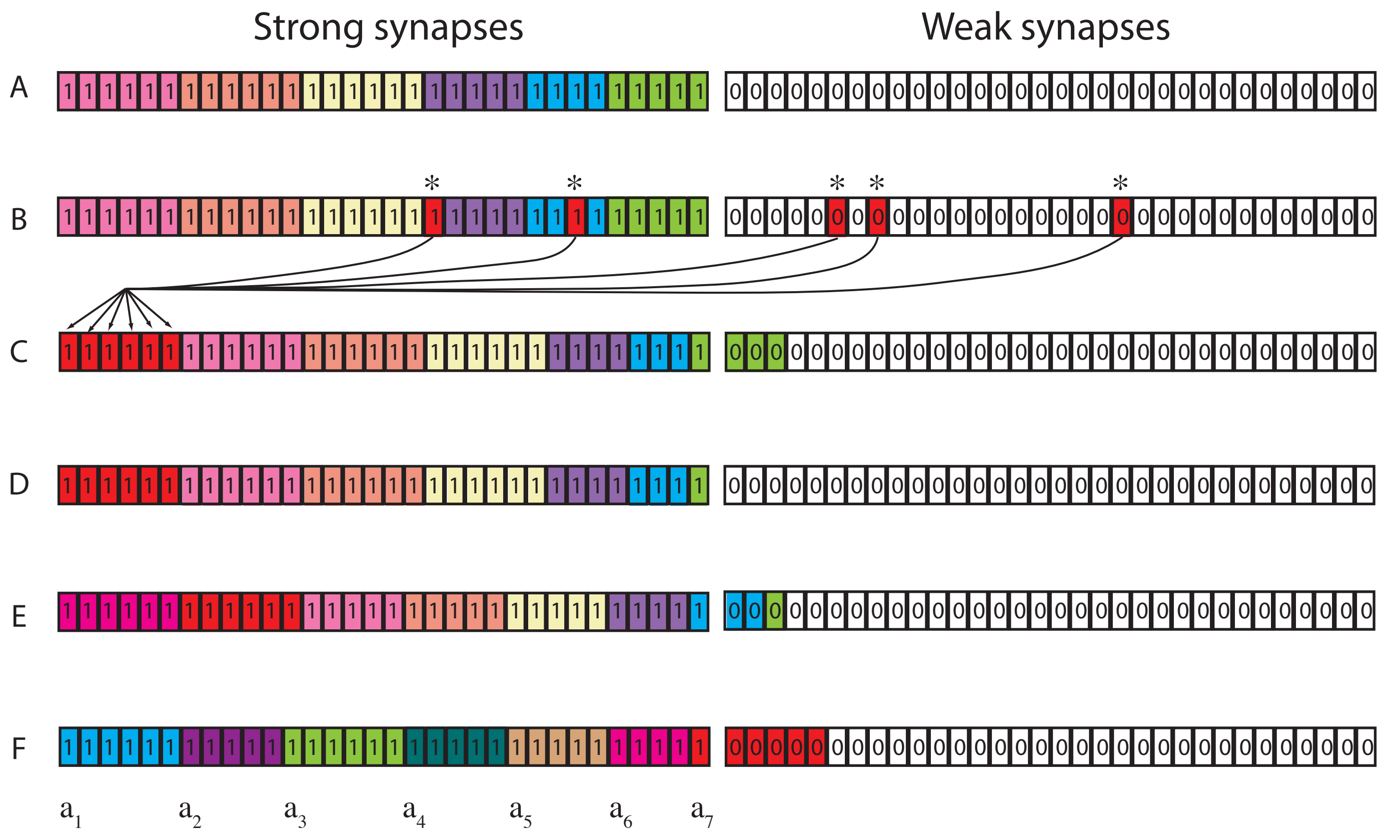
Strong synapses
Weak synapses
A
B
C
D
E
F
a1
a2
a3
a4
a5
a6
a7

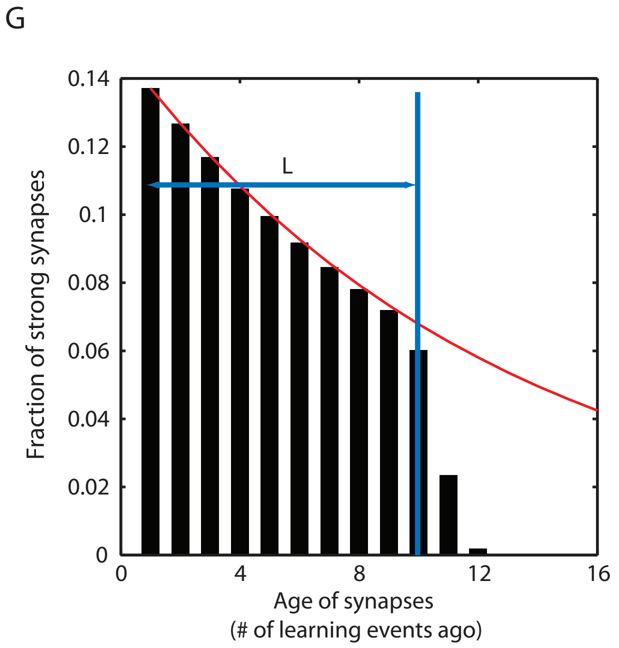
G
L
Fraction of strong synapses
Age of synapses
(# of learning events ago)

Figure 3.
