# Supplementary material for: How Dendrites Affect Online Recognition Memory
Source: PLoS Comput Biol. 2019 May 3;15(5):e1006892. doi: 10.1371/journal.pcbi.1006892 (PMC6527246; doi:10.1371/journal.pcbi.1006892)
Supplement: S1 Data — (ZIP) [file pcbi.1006892.s002.zip › Plos data:code/Figure data.zip!/Figure data/old/2.pdf]

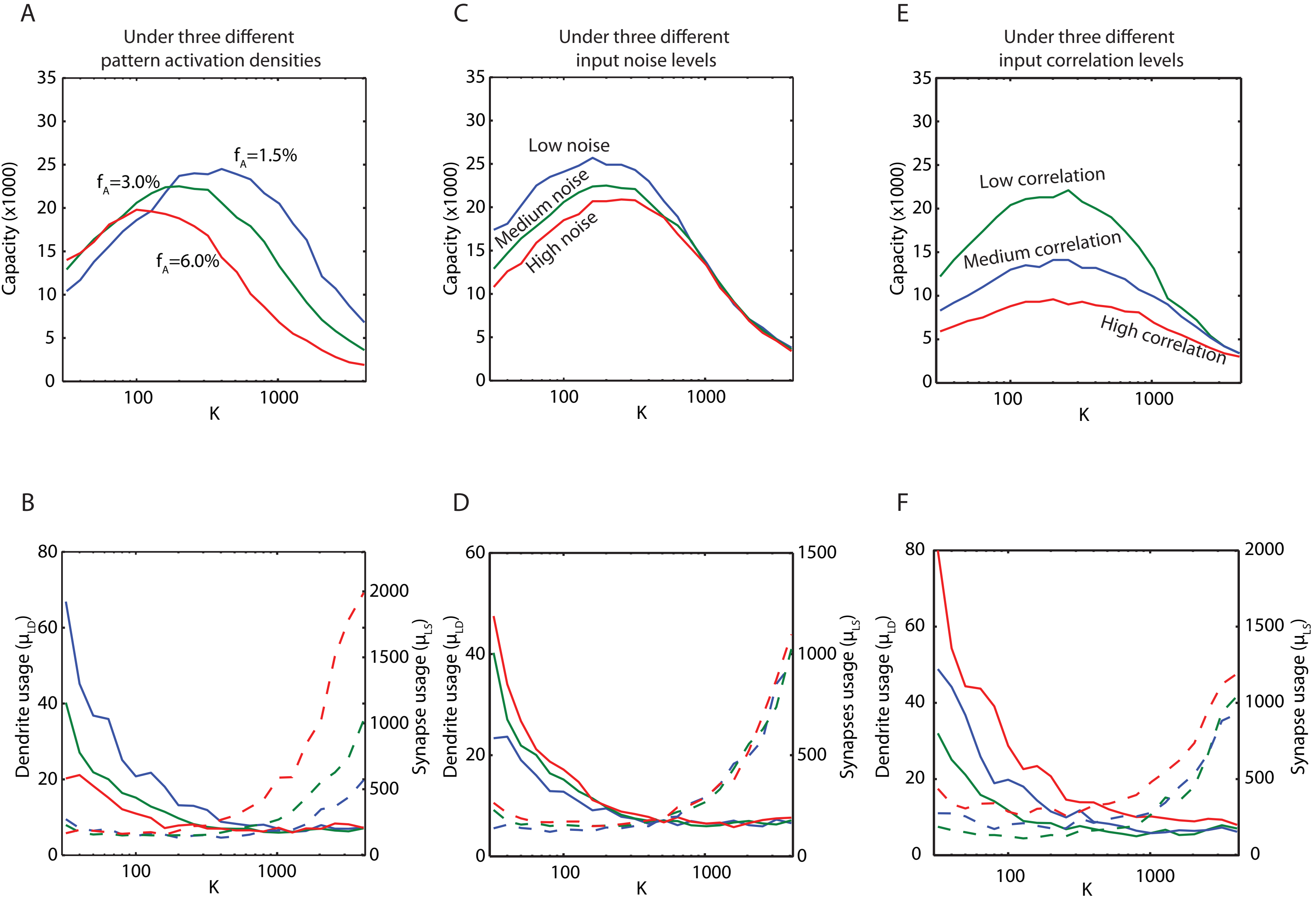

A
Under three different
pattern activation densities
Capacity (x1000)
f_A=1.5%
f_A=3.0%
f_A=6.0%
K
C
Under three different
input noise levels
Low noise
Medium noise
High noise
E
Under three different
input correlation levels
Low correlation
Medium correlation
High correlation
B
Dendrite usage (μ_{LD})
Synapse usage (μ_{LS})
D
Synapses usage (μ_{LS})
F

Figure 2
